# Chlorinated Benzo[1,2‐b:4,5‐c′]dithiophene‐4,8‐dione Polymer Donor: A Small Atom Makes a Big Difference

**DOI:** 10.1002/advs.202003641

**Published:** 2021-01-04

**Authors:** Pengjie Chao, Hui Chen, Mingrui Pu, Yulin Zhu, Liang Han, Nan Zheng, Jiadong Zhou, Xiaoyong Chang, Daize Mo, Zengqi Xie, Hong Meng, Feng He

**Affiliations:** ^1^ Shenzhen Grubbs Institute and Department of Chemistry Southern University of Science and Technology Shenzhen 518055 China; ^2^ School of Advanced Materials Peking University Shenzhen Graduate School Peking University Shenzhen 518055 China; ^3^ Academy for Advanced Interdisciplinary Studies and Department of chemistry Southern University of Science and Technology Shenzhen 518055 China; ^4^ Institute of Polymer Optoelectronic Materials and Devices State Key Laboratory of Luminescent Materials and Devices South China University of Technology Guangzhou 510640 China; ^5^ Guangdong Provincial Key Laboratory of Catalysis Southern University of Science and Technology Shenzhen 518055 China

**Keywords:** benzo[1,2‐b:4,5‐c']dithiophene‐4,8‐dione, chlorinated donors, chlorination, polymer solar cells

## Abstract

The position of a chlorine atom in a charge carrier of polymer solar cells (PSCs) is important to boost their photovoltaic performance. Herein, two chlorinated D‐A conjugated polymers PBBD‐Cl‐α and PBBD‐Cl‐β are synthesized based on two new building blocks (TTO‐Cl‐α and TTO‐Cl‐β) respectively by introducing the chlorine atom into α or β position of the upper thiophene of the highly electron‐deficient benzo[1,2‐b:4,5‐c′]dithiophene‐4,8‐dione moiety. Single‐crystal analysis demonstrates that the chlorine‐free TTO shows a π‐π stacking distance (*d*_π‐π_) of 3.55 Å. When H atom at the α position of thiophene of TTO is replaced by Cl, both π‐π stacking distance (*d*_π‐π_ = 3.48 Å) and Cl···S distance (*d*_Cl‐S_ = 4.4 Å) are simultaneously reduced for TTO‐Cl‐α compared with TTO. TTO‐Cl‐β then showed the Cl···S non‐covalent interaction can further shorten the intermolecular π‐π stacking separation to 3.23 Å, much smaller than that of TTO‐Cl‐α and TTO. After blending with BTP‐eC9, PBBD‐Cl‐β:BTP‐eC9‐based PSCs achieved an outstanding power conversion efficiency (PCE) of 16.20%, much higher than PBBD:BTP‐eC9 (10.06%) and PBBD‐Cl‐α:BTP‐eC9 (13.35%) based devices. These results provide an effective strategy for design and synthesis of highly efficient donor polymers by precise positioning of the chlorine substitution.

## Graphical abstract

Chlorination of the β‐position of benzo[1,2‐b:4,5‐c′]dithiophene‐4,8‐dione can enhance the intermolecular interaction. Single‐crystal analysis demonstrates that TTO‐Cl‐β exhibits the smallest π‐π stacking distance of 3.23 Å, much smaller than that of TTO‐Cl‐α and TTO. Accordingly, PBBD‐Cl‐β based on TTO‐Cl‐β achieved an outstanding power conversion efficiency (PCE) of 16.20%, providing a new insight for the design of acceptor units.

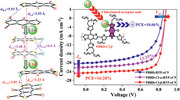

Polymer solar cells (PSC) with a bulk heterojunction (BHJ) have attracted considerable attention owing to their unique advantages which include low toxicity, light weight, semitransparency, and large‐area flexible solar panels that can be fabricated by cost‐effective solution processing techniques.^[^ ^1^, ^2^, ^3^, ^4^, ^5^, ^6^, ^7^ ^]^ Recently, benefiting from the rapid progress of new photovoltaic materials, especially fused‐ring electron acceptors (FREA), single‐junction PSCs have made a remarkable breakthrough.^[^ ^8^, ^9^, ^10^, ^11^, ^12^, ^13^ ^]^ and much recent work based on FREA single‐junction PSCs has increased the power conversion efficiency (PCE) up to 18%,^[^ ^14^, ^15^ ^]^ demonstrating an optimistic prospect and great potential for commercial application.^[^ ^14^, ^15^, ^16^, ^17^, ^18^, ^19^, ^20^ ^]^ These encouraging advances have shown that designing new organic photovoltaic materials is one of the most effective strategies to achieve an excellent PCE for PSCs.^[^ ^21^, ^22^, ^23^, ^24^ ^]^

Amongst donor materials for high solar conversions, the donor–acceptor (D–A) type of conjugated polymers, especially with electron‐withdrawing halogen substituents, have been shown to be effective. The halogenation of D–A type of conjugated polymers has various positive impacts on their optoelectronic properties, energy levels, and the planarity and crystallinity due to the non‐covalent halogen bonding.^[^ ^7^, ^25^, ^26^, ^27^ ^]^ Among all of halogen elements, fluorine was the most widely used to fine‐tune polymer properties and was very successful in increasing the efficiency of polymer solar cells. Despite these achievements of fluorinated materials, the fluorine exchange reactions always delivered a low yield and involved toxic intermediates. The final materials were therefore expensive and not the best choice for expansion of industrial mass production.^[^ ^28^, ^29^, ^30^, ^31^, ^32^ ^]^ On account of the low cost of raw materials, chlorination has become more important in the last 3 years as a means of improving photovoltaic performance.^[^ ^33^, ^34^ ^]^ However, research into chlorinated donor polymers lagged behind that of the fluorine‐substituted counterparts.^[^ ^25^, ^35^, ^36^ ^]^ Although less thoroughly investigated, chlorinated donor polymers exhibit outstanding photovoltaic performance and have achieved very great success.^[^ ^16^, ^37^ ^]^ For example, using a chlorinated polymer donor PBBSe‐Cl, we recently achieved a high PCE of 14.4% for single‐junction PSCs.^[^ ^38^ ^]^ Yan et al. continually realized a record PCE of 17% when they paired Y6 with the chlorinated polymer PM7, which has chlorine substituents in the donor moiety of this D–A type material.^[^ ^14^, ^17^ ^]^ Previous reports showed that chlorination of the thiophene side chain of benzo[1,2‐b:4,5‐b´]dithiophene (BDT) could simultaneously improve the open‐circuit voltage (*V*_oc_), the short‐circuit current density (*J*_sc_) and the fill factor (FF).^[^ ^10^, ^33^, ^39^, ^40^, ^41^, ^42^ ^]^ Chlorination of the polymer backbone increases *V*_oc_ but makes less of a contribution to *J*_sc_.^[^ ^37^, ^43^, ^44^ ^]^ This can be attributed to the large atomic radius of chlorine, which causes steric hindrance and decreases molecular planarity when a Cl atom is introduced directly to the conjugated backbone.^[^ ^34^ ^]^ In addition, there are relatively few chlorinated D–A type donor polymers with a Cl atom in the acceptor moiety, and such devices show PCEs of only ≈12%.^[^ ^37^, ^44^, ^45^ ^]^ It is therefore urgent to develop higher performance donors with a chlorine substituted acceptor moiety. These can help us to explore the effect of chlorination at different positions of the D–A polymers, and reveal the crucial principles that enable the design of low cost and highly efficient chlorinated photovoltaic materials.

In addition to halogenation to control the intermolecular interactions and film morphologies, the backbone design is of great importance to adjust the optical absorption and charge carrier transport for efficient solar conversion. Recently, we showed that 5,7‐dibromo‐2,3‐bis(2‐ethylhexyl)benzo[1,2‐b:4,5‐c**′**]dithiophene‐4,8‐ dione (TTDO) formed by combining cyclohexane‐1,4‐dione with a thio[3,4‐b]thiophene unit (TT), has a strong electron‐deficient capability and the resulted TTDO acceptor moiety shows better molecular overlap and a shorter *π*—*π* stacking distance between adjacent molecules than its isomer BDTO which lacks the TT skeleton.^[^ ^21^ ^]^ The question was how to further control the intermolecular interaction of this electron‐deficient acceptor unit. Inspired by the chlorination strategy detailed above, we designed and synthesized a series of chlorinated benzo[1,2‐b:4,5‐c**′**]dithiophene‐4,8‐dione (TTO‐Cl‐*α* and TTO‐Cl‐*β*) acceptor units which promised improved charge carrier transport by enhancing intermolecular interactions from the introduction of Cl atoms. Unlike the conventional chlorine‐substituted polymers, in which the chlorine is directly introduced into the backbone of the acceptor unit, the position of chlorination in these two materials is located on the upper thiophene (*α‐* or *β*‐position) of benzo[1,2‐b:4,5‐c**′**]dithiophene‐4,8‐dione unit, which is some distance from the backbone in the corresponding polymers. This could weaken the distortion of the polymer backbone caused by the large Cl atoms, and mainly utilizes the non‐covalent interactions of Cl to optimize the molecular arrangement in the solid state. The single‐crystal analysis demonstrates that the chlorine‐free TTO showed the *π*—*π* stacking distance (*d*_*π*—*π*_) to be 3.55 Å, and the H···S separation (*d*_H—S_) is 5.93 Å. When *α*‐H of TTO was replaced by Cl, both *π*—*π* stacking distance (*d*_*π*—*π*_ = 3.48 Å) and Cl···S distance (*d*_Cl—S_ = 4.4 Å) were simultaneously reduced for TTO‐Cl‐*α* compared with that of TTO. The chlorine atoms in two adjacent TTO‐Cl‐*α* molecules tended to line up on the same side, which will form two cross Cl···S interactions and decrease the *π*—*π* stacking distance in some degree. But the arrangement of Cl atoms in this case cannot avoid steric hindrance from the two homolateral chlorine atoms, and the Cl‐substituted position should be further optimized targeting much closer *π*—*π* stacking. And when *β*‐H of TTO was replaced by Cl, the chlorine atoms in two adjacent TTO‐Cl‐*β* molecules were arranged at both sides, which could avoid the steric hindrance from two closed chlorine atoms. In this case, TTO‐Cl‐*β* showed that the Cl···S non‐covalent interaction can further shorten the intermolecular *π*—*π* stacking separation to ≈3.23 Å, which is much smaller than that of TTO‐Cl‐*α* and TTO molecules and benefit the charge carrier hopping transport in final polymers. As the result, one of the chlorinated polymers, PBBD‐Cl‐*β*, which is based on TTO‐Cl‐*β* achieved an outstanding PCE of 16.20% when combined with a fused ring acceptor BTP‐eC9. As a comparison, the chlorine‐free and *α*‐position Cl‐substituted counterpart polymers, PBBD and PBBD‐Cl‐*α*, were also prepared and both devices were found to give a relatively low efficiency of 10.06% and 13.35%. This result indicates that the chlorination of the highly electron‐deficient benzo[1,2‐b:4,5‐c**′**]dithiophene‐4,8‐dione core at precise position could greatly improve the final molecular stacking, rendering these type of materials good candidates for organic photovoltaics. With its impact within the solar cell research community, it also provides a new insight enabling the design high‐performance polymer donors with electron‐deficient construction units, especially fine adjustment of the intermolecular interactions and film morphologies dominated by the effect of non‐covalent chlorination.

The synthetic routes to the polymers PBBD, PBBD‐Cl‐*α*, and PBBD‐Cl‐*β* are shown in **Scheme** **1**, and the corresponding procedure is detailed in the Supporting Information (SI). Compounds (3) and M4 were purchased from chemical suppliers. The monomers M1, M2, and M3 were synthesized by published methods.^[^ ^21^ ^]^ PBBD PBBD‐Cl‐*α* and PBBD‐Cl‐*β* have the number‐average molecular weights (*M*_n_) 30.60, 33.52, and 31.07 kDa, and corresponding polydispersity indexes of 2.16, 2.36, and 1.83, respectively. Three polymers are freely soluble in chlorobenzene (CB) and possess good thermal stability with a decomposition temperature (*T*_d_) around 400 °C with a 5% weight loss (Figure S2a, Supporting Information). As exhibited in Figure S2b, Supporting Information, they have no endo‐ and exothermal peaks in the differential scanning calorimetry curve from 50 to 300 °C, indicating the strong rigidity of the polymer main‐chain.

**Scheme 1:**
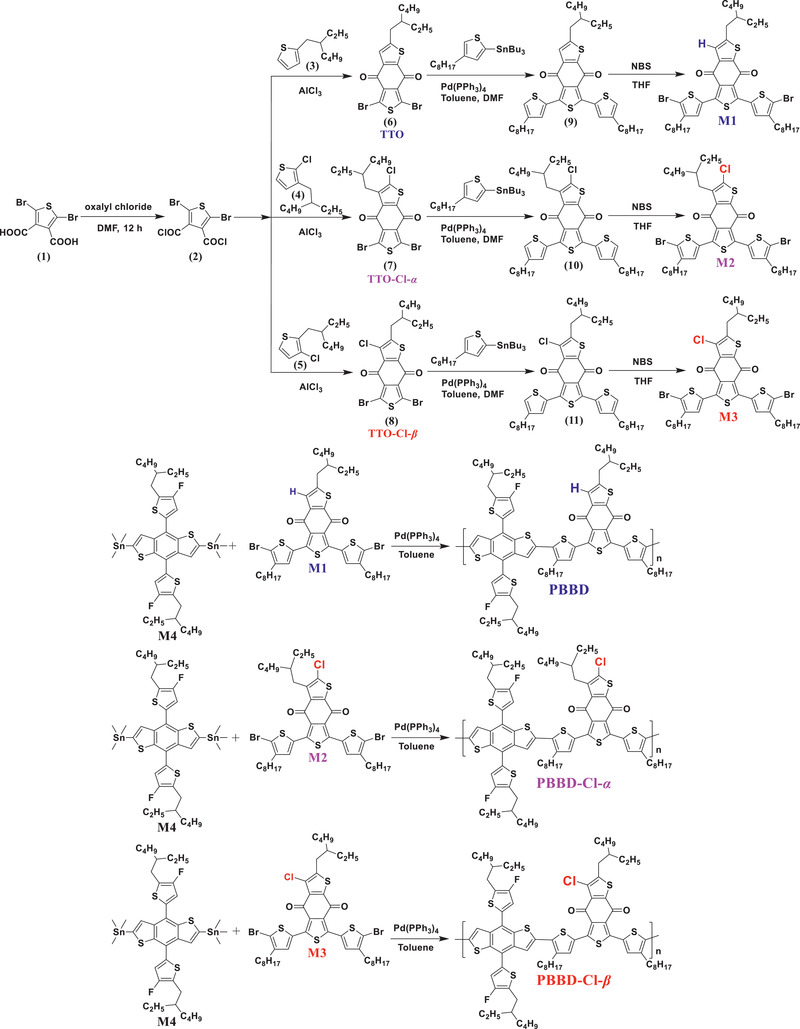
Synthesis of monomers (TTO, TTO‐Cl‐*α*, TTO‐Cl‐*β*, M1, M2 and M3) and polymers (PBBD, PBBD‐Cl‐*α* and PBBD‐Cl‐*β*).

In order to investigate the influence of chlorination on the structure‐property relationships, especially the effect of chlorine on the intermolecular interactions, single‐crystals of TTO, TTO‐Cl‐*α*, and TTO‐Cl‐*β* were grown and their diffraction data were determined by X‐ray single crystal diffraction. The single‐crystal structures of TTO, TTO‐Cl‐*α*, and TTO‐Cl‐*β* are depicted in **Figure** **1**. As displayed in Figure 1a, the side view of chlorine‐free TTO showed the *π*—*π* stacking distance (*d*_*π*—*π*_) to be 3.55 Å, and the H···S separation (*d*_H—S_) is 5.93 Å. The top view in Figure 1b shows the intermolecular packing extending along the molecular long axis with an overlap corresponding to a single thiophene ring area. Cl atom can accept the *π*‐electron and lone pair electron of heteroatoms to form the intermolecular Cl···S and Cl···*π* non‐covalent interaction because of the unoccupied 3d orbitals.^[^ ^13^ ^]^ As exhibited from the side view of TTO‐Cl‐*α* in Figure 1c, both *π*—*π* stacking distance (*d*_*π*—*π*_ = 3.48 Å) and Cl···S distance (*d*_Cl—S_ = 4.4 Å) were simultaneously reduced for TTO‐Cl‐*α* compared with that of TTO, which should be attributed to the weak *π*—*π* stacking and Cl···S non‐covalent interaction in TTO‐Cl‐*α*. Besides, as displayed from the top view of TTO‐Cl‐*α* in Figure 1d, in comparison with TTO, TTO‐Cl‐*α* molecules were arranged side by side along the molecular short axis and the alkyl chains are all facing outward; meanwhile, due to the distinct steric repulsion effect of Cl atoms at same side, TTO‐Cl‐*α* slipped significantly in the direction of the short axis of the molecule, and this kind of molecular arrangement should be maintained by weak *π*—*π* stacking and Cl···S non‐covalent interlocking interaction. Furthermore, as shown in Figure 1e, the intermolecular arrangement for TTO‐Cl‐*β* exhibited a clear change again when the *β*‐position H atom of thiophene of TTO was replaced by Cl. First, the distance (*d*_H—S_ = 5.93 Å) between H and S in the TTO unit was reduced to the new distance (*d*_Cl—S_ = 3.81 Å) between Cl and S in the TTO‐Cl‐*β* unit, indicating very strong Cl···S and Cl···*π* non‐covalent interaction existed in TTO‐Cl‐*β*. In addition, precisely because of this very strong Cl···S and Cl···*π* non‐covalent interaction, the single‐crystal structure of TTO‐Cl‐*β* achieved the much smaller *π*—*π* stacking distance (*d*_*π*—*π*_) of 3.23 Å compared with that in TTO (3.55 Å), demonstrating that TTO‐Cl‐*β* possesses the smaller steric repulsion and tighter molecular packing. As exhibited in Figure 1d, one of two TTO‐Cl‐*β* molecules has rotated through ≈90° but still maintains almost the same area overlap with TTO which should be attributed to the smaller steric repulsion and the strong Cl···S and Cl···*π* non‐covalent interaction. Based on these results, the single‐crystal structures of TTO, TTO‐Cl‐*α*, and TTO‐Cl‐*β* directly demonstrate the existence of strong Cl···S non‐covalent interaction, and a strong Cl···S and Cl···*π* non‐covalent interaction can significantly enhance the intermolecular *π*—*π* stacking effect. Herein, these results indicated both chlorination and suitable position of chlorination played a significant role in molecular arrangement.

**Figure 1:**
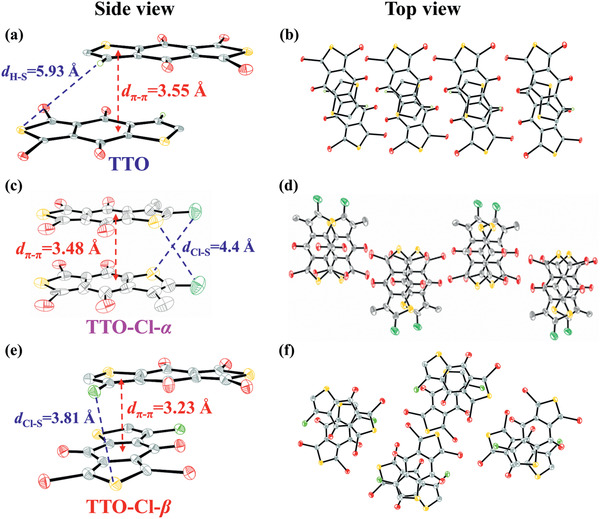
Single‐crystal structure: a) side view of TTO, b) top view of TTO, c) side view of TTO‐Cl‐*α* and d) top view of TTO‐Cl‐*α* e) side view of TTO‐Cl‐*β*, and f) top view of TTO‐Cl‐*β*. Note: here the alkyl chain (2‐ethylhexyl) were omitted for clarity.

To gain more insight into role of chlorine in the polymer, theoretical calculations by density functional theory at the B3LYP/6‐31G(d,p) level were performed to investigate the influence of chlorine on the molecular conformations and energy levels. In the simulation calculations, the long alkyl side chains were replaced with methyl groups and, as exhibited in Figure S3, Supporting Information, the two dihedral angles on both sides of the thiophene *π*‐bridge are 23.40° and 12.60° for PBBD, which are larger than those of PBBD‐Cl‐*β* (20.41° and 7.89°). Accordingly, PBBD‐Cl‐*β* is more planar than PBBD, as can be seen in the side view. This result indicates that chlorination of the acceptor unit at a position distant from the polymer backbone not only results in steric hindrance, but also improves the molecular planarity. The highest occupied molecular orbital (HOMO) and lowest unoccupied molecular orbital (LUMO) levels for PBBD and PBBD‐Cl‐*β* are −5.04/−2.62 eV and −5.10/−2.72 eV, respectively. In addition, as shown in Figure S3, Supporting Information, the HOMO levels are distributed along the entire polymeric backbone, while the LUMO levels are located entirely in the acceptor unit.

The UV–vis absorption spectra of donor polymers PBBD, PBBD‐Cl‐*α* and PBBD‐Cl‐*β* in chlorobenzene are shown in Figure S4, Supporting Information. Polymer PBBD exhibits the maximum absorption at 575 nm with a molar extinction coefficient of 6.1 × 10^4^ m ^−1^ cm^−1^(**Table** **1**), and as shown in **Figure** **2**a, PBBD film gives an absorption band red‐shifted absorption by 21 nm from that in solution. Compared with PBBD, chlorinated polymers PBBD‐Cl‐*α* and PBBD‐Cl‐*β* exhibit red‐shifted maximum absorption at 601 and 608 nm with higher molar extinction coefficient of 7.7 × 10^4^ and 8.9 × 10^4^ m ^−1^ cm^−1^, respectively, implying a stronger intramolecular charge transfer (ICT) and effective electron delocalization of PBBD‐Cl‐*α* and PBBD‐Cl‐*β* after introducing Cl atom. PBBD film absorption was red‐shifted by 21 nm when compared with that in solution, while the red‐shifted values for both PBBD‐Cl‐*α* and PBBD‐Cl‐*β* film are only 5 nm with the weak shoulder peak. This indicates that a stronger aggregation behavior for PBBD‐Cl‐*α* and PBBD‐Cl‐*β* is formed in chlorobenzene solution, which is beneficial to tuning the film morphology of photovoltaic layer. Due to the inductive effect of Cl, the introduction of Cl atom could enhance the ICT effect, leading to the red‐shifted maximum absorption peak for PBBD‐Cl‐*β* compared to non‐chlorinated polymer PBBD. In addition, the single crystal structure of TTO‐Cl‐*β* achieved the much smaller *π*—*π* stacking distance (*d*_*π*—*π*_) of 3.23 Å because of the stronger Cl···S and Cl···*π* non‐covalent interaction compared with that in TTO (3.55 Å), which can increase the *π*—*π* stacking and aggregation effect of PBBD‐Cl‐*β*, resulted in the red‐shifted maximum absorption peak of PBBD‐Cl‐*β*. As displayed in Figure S5, Supporting Information, PBBD‐Cl‐*α* film shows a higher absorption coefficient of 4.4 × 10^4^ cm^−1^ than the PBBD film (3.8 × 10^4^ cm^−1^), and PBBD‐Cl‐*β* possesses the highest absorption coefficient of 5.0 × 10^4^ cm^−1^ among three polymer films, implying that the PBBD‐Cl‐*β* film has the strongest light‐harvesting capability. The optical band gaps ($E_{g}^{\mathrm{opt}}$), determined by the absorption onset for PBBD, PBBD‐Cl‐*α*, and PBBD‐Cl‐*β* are 1.76, 1.79, and 1.78 eV respectively.

**Table 1:** Photophysical properties and energy levels of the polymers

Polymer	Solution		Film		$E_{g}^{\mathrm{opt}}$ [eV]	HOMO [eV]	LUMO [eV]
	*λ*_max_ [nm]	Ε [M^−1^ cm^−1^]	*λ*_max_ [nm]	*λ*_edge_ [nm]			
PBBD	575	6.1 × 10^4^	596	705	1.76	−5.24	−3.48
PBBD‐Cl‐*α*	601	7.7 × 10^4^	606	692	1.79	−5.33	−3.54
PBBD‐Cl‐*β*	608	8.9 × 10^4^	613	694	1.78	−5.33	−3.55

**Figure 2:**
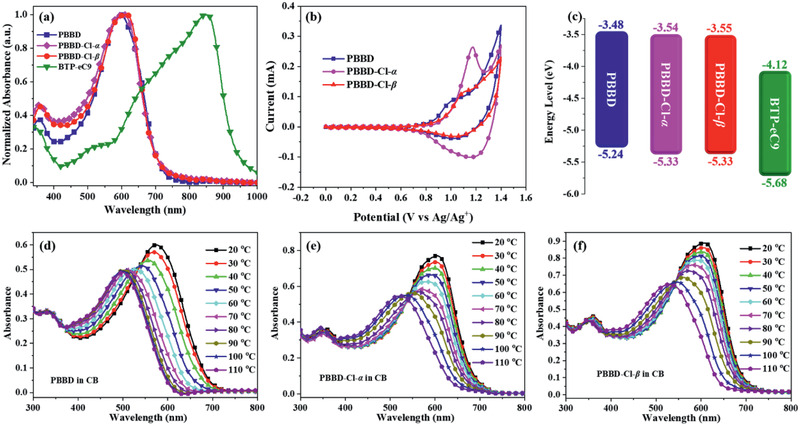
a) Normalized UV–vis absorption spectra of polymer films. b) CV curves and c) energy level diagrams for PBBD, PBBD‐Cl‐*α*, PBBD‐Cl‐*β*, and BTP‐eC9. d) Temperature‐dependent absorption spectra for PBBD in chlorobenzene (CB). e) Temperature‐dependent absorption spectra for PBBD‐Cl‐*α* in CB. f) Temperature‐dependent absorption spectra for PBBD‐Cl‐*β* in CB.

The HOMO levels of PBBD, PBBD‐Cl‐*α*, and PBBD‐Cl‐*β* were determined by cyclic voltammetry and shown in Figure 2b. The onset oxidation potential were determined to be 0.84, 0.93, and 0.93 eV for PBBD, PBBD‐Cl‐*α*, and PBBD‐Cl‐*β*, respectively. As exhibited in Figure 2c and Table 1, PBBD shows a HOMO level of −5.24 eV, while both PBBD‐Cl‐*α* and PBBD‐Cl‐*β* display deeper HOMO levels of −5.33 eV, suggesting higher *V*_oc_ for PBBD‐Cl‐*α*‐ and PBBD‐Cl‐*β*‐based devices. The temperature‐dependent absorption of PBBD (Figure 2d), PBBD‐Cl‐*α* (Figure 2e) and PBBD‐Cl‐*β* (Figure 2f) in CB solution was also performed to investigate the aggregation behavior for three polymers. PBBD shows a blue‐shifted tendency with a synchronously decreased absorbance value when the temperature is increased from 20 to 60 °C, and the absorption spectra, including the absorption edge and absorption intensity almost no longer changes when the temperature exceeds 70 °C, indicating PBBD is already been thoroughly disaggregated in a single molecular state in solution at 70 °C. Moreover, as exhibited in Figure 2e, PBBD‐Cl‐*α* also yields the gradually blue‐shifted absorption edges and decreased absorption value when increasing the temperature from 20 to 70 °C, and when the temperature continues to rise, the absorbance value no longer changes significantly, but the onset absorption gradually blue shifts, indicating PBBD‐Cl‐*α* achieves a stronger aggregation than PBBD in CB solution because of the Cl···S non‐covalent interlocking interaction. Furthermore, the blue‐shifted tendency and decreased absorption intensity of PBBD‐Cl‐*β* are observed throughout the entire heating process from 20 to 110 °C, and before the temperature rises to 90 °C, the blue‐shifted trend of absorption spectra for PBBD‐Cl‐*β* is much slower than that of PBBD‐Cl‐*α*. This demonstrates PBBD‐Cl‐*β* has a much more strongly aggregated than PBBD‐Cl‐*α*, that is to say, PBBD‐Cl‐*β* exhibits the strongest aggregation behavior among three polymers. This should be attributed to the tightest *π*—*π* stacking and strongest Cl···S and Cl···*π* non‐covalent interaction in TTO‐Cl‐*β*, indicating that the rational position chlorination can enhance the intermolecular *π*—*π* interactions and aggregation behavior.

For the fused core 5,7‐dibromo‐2,3‐bis(2‐ethylhexyl)benzo[1,2‐b:4,5‐c**′**]dithiophene‐4,8‐dione (TTDO), TTDO has two 2‐ethylhexyl alkyl chains onto the upper thiophene. The single crystal structure of TTDO gave a *π*—*π* stacking distance (*d*_*π*—*π*_) of 3.43 Å, and polymer PBBTT‐F exhibited a strong aggregation.^[^ ^21^ ^]^ Compared with TTDO, TTO just has one 2‐ethylhexyl alkyl chain and its single crystal obtained a little larger *d*_*π*—*π*_ of 3.55 Å. This could be likely that TTDO slips in the same direction and maintains the edge‐to‐edge molecular arrangement, avoiding the repulsion of alkyl chains; however, for TTO, it exhibited a face‐to‐face cross arrangement, leading to the obvious mutual repulsion of alkyl chains, resulting in a larger *π*—*π* stacking distance. Consequently, the corresponding polymer PBBD showed a poor molecular aggregation determined from temperature‐dependent absorption. When the H atom at *α* position of thiophene of TTO was replaced by Cl, TTO‐Cl‐*α* displayed a decreased *d*_*π*—*π*_ = 3.48 Å with a Cl···S distance of 4.4 Å (*d*_Cl—S_) compared with that of TTO. Meantime, TTO‐Cl‐*α* were arranged side by side and slipped significantly due to the distinct steric repulsion effect of Cl atoms. TTO‐Cl‐*α* displayed a similar edge‐to‐edge arrangement with TTDO, and polymer PBBD‐Cl‐*α* achieved slightly weaker aggregation than PBTT‐F, which should be attributed to the steric hindrance of alkyl chain (—C_8_H_17_) onto the polymer backbone.^[^ ^46^, ^47^ ^]^ Furthermore, the single crystal of TTO‐Cl‐*β* exhibited a much smaller *d*_*π*—*π*_ of 3.23 Å and *d*_Cl—S_ = 3.81 Å because of the smaller steric repulsion, and stronger Cl···S and Cl···*π* non‐covalent interaction existed in TTO‐Cl‐*β* when the *β*‐position H atom of thiophene of TTO was replaced by Cl. Accordingly, polymer PBBD‐Cl‐*β* exhibited a very strong intermolecular aggregation even though the ‐C_8_H_17_ alkyl chain existed onto the polymer backbone which could help to improve charge transport.

To evaluate the photovoltaic performance of PBBD, PBBD‐Cl‐*α*, and PBBD‐Cl‐*β*, the acceptor BTP‐eC9 was blended with PBBD, PBBD‐Cl‐*α* and PBBD‐Cl‐*β* in sequence to fabricate the BHJ PSCs. The device was constructed with a conventional architecture of ITO/PEDOT:PSS/polymer:BTP‐eC9/PNDIT‐F3N/Ag. Herein, chlorobenzene (CB) was selected as the processing solvent and the active layer was obtained by spin coating with a weight ratio of 1:1.2 (w:w), and annealed at 100 °C. Their current density–voltage (*J*–*V*) profiles were shown in **Figure** **3**a and the corresponding photovoltaic data were summarized in **Table** **2**. The PBBD‐based device exhibits a low PCE of 10.06% with a *V*_oc_ of 0.84 V. Furthermore, after introducing Cl onto the *α* position of thiophene, PBBD‐Cl‐*α*:BTP‐eC9‐based device obtained an elevated PCE of 13.35%, with a synchronously improved *V*_oc_ of 0.87 V, a *J*_sc_ of 22.08 mA cm^−2^, and an *FF* of 69.35%. However, when introducing Cl onto the *β* position of thiophene, the photovoltaic performance of PBBD‐Cl‐*β*:BTP‐eC9‐based device was further improved, and achieved an outstanding PCE of 16.20% and a high *V*_oc_ of 0.87 V, with a simultaneously increased *J*_sc_ of 24.59 mA cm^−2^, and an FF of 75.53% compared with that of PBBD‐Cl‐*α*. The improved *V*_oc_ value for PBBD‐Cl‐*α*:BTP‐eC9‐ and PBBD‐Cl‐*β*:BTP‐eC9‐based device should be attributed to the deeper HOMO level of PBBD‐Cl‐*α* and PBBD‐Cl‐*β*. The increased *J*_sc_ value PBBD‐Cl‐*α*:BTP‐eC9‐ and PBBD‐Cl‐*β*:BTP‐eC9‐based device should be assigned to the gradually enhanced light‐harvesting ability for PBBD‐Cl‐*α* and PBBD‐Cl‐*β*. Figure 3b shows the external quantum efficiency (EQE) spectra of the corresponding PSCs. The PBBD‐based device showed a maximum EQE value of 65% at 490 nm. In addition, PBBD‐Cl‐*α*:BTP‐eC9‐based device gave a much higher maximum EQE value of 72% at 640 nm. Distinctly, the device based on PBBD‐Cl‐*β*:BTP‐eC9 achieved the strongest photoresponse with a maximum EQE value of 80% at 636 nm among the three devices, indicating the efficient photocurrent generation of the PBBD‐Cl‐*β*:BTP‐eC9‐device. The integrated current densities from the EQE spectra of the three devices are 19.32, 21.12, and 23.50 mA cm^−2^, respectively, in good agreement with the results of the *J*–*V* measurements.

**Figure 3:**
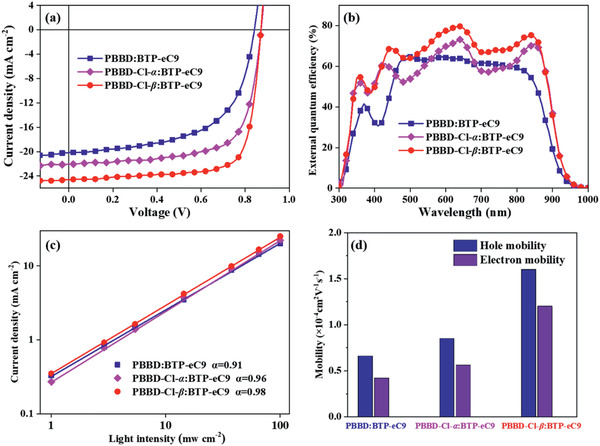
a) *J*−*V* curves; b) EQE curves; c) *J*_sc_ versus the light intensity; d) hole and electron mobility of the optimized blend films.

**Table 2:** Optimized photovoltaic parameters of the PSCs for devices based on PBBD, PBBD‐Cl‐*α* and PBBD‐Cl‐*β*

Polymers	*V*_oc_ [V]	*J*_sc_ [mA cm^−2^]	*J*_sc_ ^a)^ [mA cm^−2^]	FF [%]	PCE^b)^ [%]
PBBD	0.84	20.15	19.32	59.28	10.06 (9.86 ± 0.18)
PBBD‐Cl‐*α*	0.87	22.08	21.12	69.35	13.35 (13.19 ± 0.19)
PBBD‐Cl‐*β*	0.87	24.59	23.50	75.53	16.20 (15.92 ± 0.30)

^a)^ *J*_sc_ calculated from the EQE

^b)^ Average values with standard deviations are based on 15 devices.

The correlation of light intensity (*P*_light_) and *J*_sc_ (*J*_sc_∝*P*_light_^*α*^) was also measured to investigate the charge recombination of the relevant PSCs. As shown in Figure 3c, the fitted *α* values for PBBD:BTP‐eC9, PBBD‐Cl‐*α*:BTP‐eC9, and PBBD‐Cl‐*β*:BTP‐eC9 devices are 0.91, 0.96, and 0.98, respectively, suggesting the least bimolecular recombination for PBBD‐Cl‐*β*:BTP‐eC9, which serves to enhance *J*_sc_ and FF. Hole and electron mobilities were also measured with the space‐charge limited current method, as shown in Figures S6–S8, Supporting Information and Figure 3d. As displayed in Figure S6, Supporting Information, the hole mobility of the PBBD, PBBD‐Cl‐*α*, and PBBD‐Cl‐*β* neat films were 9.8 × 10^−5^, 5.2 × 10^−4^, and 8.6 × 10^−4^ cm^2^ v^−1^ s^−1^, respectively. Meantime, the hole (*μ*_h_) and electron (*μ*_e_) mobilities of PBBD:BTP‐eC9 and PBBD‐Cl‐*α*:BTP‐eC9 based devices are 6.6 × 10^−5^/4.2 × 10^−5^ and 8.5 × 10^−5^/5.6 × 10^−5^ cm^2^ v^−1^ s^−1^ (Table S3, Supporting Information), respectively. In contrast, the device based on PBBD‐Cl‐*β*:BTP‐eC9 achieved the highest hole and electron mobilities (1.6 × 10^−4^ and 1.2 × 10^−4^ cm^2^ v^−1^ s^−1^) (Table S3, Supporting Information), suggesting that the better molecular planarity of PBBD‐Cl‐*β* could contribute to the intermolecular *π*—*π* stacking. In addition, the corresponding *μ*_h_/*μ*_e_ ratios are 1.6, 1.5, and 1.3 for PBBD:BTP‐eC9, PBBD‐Cl‐*α*:BTP‐eC9 and PBBD‐Cl‐*β*:BTP‐eC9, respectively, and the more balanced and higher charge carrier transport of PBBD‐Cl‐*β*:BTP‐eC9 also accounts for its high *J*_sc_ and FF.

The surface and inner morphology of PBBD:BTP‐eC9, PBBD‐Cl‐*α*:BTP‐eC9, and PBBD‐Cl‐*β*:BTP‐eC9 blend films were investigated using atomic force microscopy (AFM) and transmission electron microscopy (TEM). As displayed in **Figure** **4**a–c, the root‐mean‐square (RMS) roughness values for PBBD:BTP‐eC9, PBBD‐Cl‐*α*:BTP‐eC9, and PBBD‐Cl‐*β*:BTP‐eC9 blend films were 3.76, 3.94, and 4.24 nm, respectively. The largest RMS value of PBBD‐Cl‐*β*:BTP‐eC9 resulted from the stronger aggregation of chlorinated PBBD‐Cl‐*β*. As shown in Figure 4d, PBBD:BTP‐eC9 blend film exhibited a small fibril‐like phase separation morphology. In addition, the PBBD‐Cl‐*α*:BTP‐eC9 film (Figure 4e) gave a cluster‐like phase separation on the basis of PBBD:BTP‐eC9 blend film. Furthermore, as exhibited in Figure 4f, when Cl atom was introduced onto the *β*‐position of the upper thiophene in TTO, the PBBD‐Cl‐*β*:BTP‐eC9 blend film shows a cluster‐like phase separation with the larger domain sizes compared with that of PBBD‐Cl‐*α*:BTP‐eC9 blend film, and polymer PBBD‐Cl‐*β* and BTP‐eC9 were mixed uniformly in solution and form a small cluster in solid state. In each cluster, PBBD‐Cl‐*β* and BTP‐eC9 were mingled uniformly which was beneficial to improve carrier splitting. The 2D interpenetrating nanofiber network morphology was also observed in the cluster structure and each cluster region was also surrounded by fibrillary networks, demonstrating the blend film of PBBD‐Cl‐*β*:BTP‐eC9 yielded a multiscale‐length morphology. Moreover, for each cluster region in the polymorphous morphology, the relatively pure and suitable phase separation morphology could contribute to exciton dissociation and charge transport, and also reduce the chance for charge recombination. In short, the chlorination of the appropriate position could form the favorable phase separation morphology, thus leading to improve *J*_sc_, FF, and realizing a high PCE.

**Figure 4:**
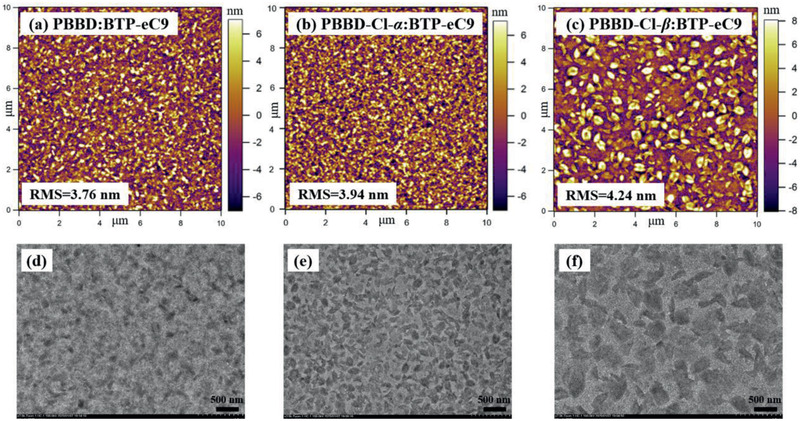
a–c) AFM height images (10 × 10 µm) and d–f) TEM images of the optimized blend films based on PBBD, PBBD‐Cl‐*α*, and PBBD‐Cl‐*β* with BTP‐eC9 in sequence.

For a better understanding of the formed cluster‐like phase separation morphology, grazing incidence wide‐angle X‐ray scattering (GIWAXS) tests were performed to investigate the molecular packing and orientation behavior in the blend films. As illustrated in **Figure** **5**a–c, the 2D‐GIWAXS profiles of all the blend films display the similar face‐on orientation in out‐of‐plane (OOP) direction, which is beneficial for vertical charge transport along the direction of electrodes. However, as exhibited in Figure 5d, a difference in the *π*—*π* stacking behaviors was apparent. First, the OOP (010) *π*—*π* stacking peak for PBBD:BTP‐eC9 blend film was located at *q*_z_ = 1.66 Å^−1^ and corresponded to the *π*—*π* stacking distance of *d*_*π*—*π*_ = 3.78 Å. In addition, the (010) *π*—*π* stacking peaks in out‐of‐plane direction for PBBD‐Cl‐*α*:BTP‐eC9 and PBBD‐Cl‐*β*:BTP‐eC9 blend films were observed at *q*_z_ = 1.69 Å^−1^ and *q*_z_ = 1.72 Å^−1^, which corresponded to the *π*—*π* stacking distances of 3.71 and 3.65Å, respectively. Obviously, the *π*—*π* stacking distance of the blend film was reduced after introducing Cl atom, and PBBD‐Cl‐*β*:BTP‐eC9 blend films exhibited the smallest *π*—*π* stacking distances among three blend films which could contribute to vertical charge transport. Besides, further investigating the crystal coherence length (CCL) of the *π*—*π* stacking in films,^[^ ^45^ ^]^ and the corresponding CCL values for PBBD:BTP‐eC9, PBBD‐Cl‐*α*:BTP‐eC9, and PBBD‐Cl‐*β*:BTP‐eC9 based blend films were 17.3, 19.8, and 20.9 Å, respectively, which is consistent with the aforementioned results of the temperature‐dependent absorption and the morphology from AFM and the TEM. It should be noted that the CCL of PBBD‐Cl‐*α*:BTP‐eC9 and PBBD‐Cl‐*β*:BTP‐eC9 based blend films were increased compared with that of PBBD based blend film, demonstrating the higher molecular order in PBBD‐Cl‐*α*:BTP‐eC9 and PBBD‐Cl‐*β*:BTP‐eC9 based blend films. Meanwhile, the higher CCL of PBBD‐Cl‐*β*:BTP‐eC9 based blend film could account for the higher and more balanced carrier mobilities in PBBD‐Cl‐*β*:‐based film. Overall, the shorter *π*—*π* stacking distances and increased CCL of PBBD‐Cl‐*β*:BTP‐eC9 based blend film deserved higher *J*_sc_, FF, and PCE of the PSCs with PBBD‐Cl‐*β* as donor, which is in good agreement with the results of *J–V* curves mentioned above.

**Figure 5:**
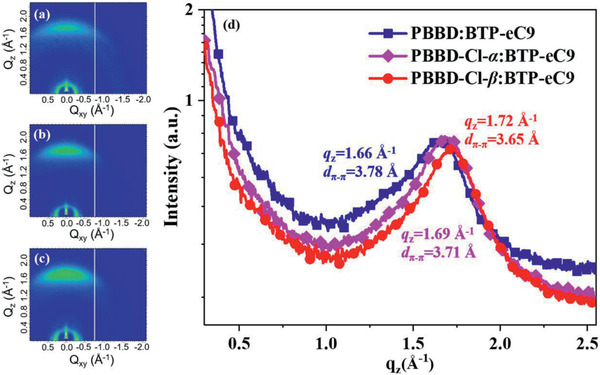
2D‐GIWAXS patterns of a) PBBD:BTP‐eC9, b) PBBD‐Cl‐*α*:BTP‐eC9, and PBBD‐Cl‐*β*:BTP‐eC9 blend film. c) Line‐cut scattering profiles out‐of‐plane of the corresponding optimized blends.

The device stability is also a key factor in evaluation of the photovoltaic performance of PSCs and consequently, the storage stability and photostability of the PBBD‐Cl‐*β*:BTP‐eC9 device were determined as shown in Figure S9, Supporting Information, and their photovoltaic parameters versus time are collected in Tables S1 and S2, Supporting Information. As displayed in Figure S9 and Table S1, Supporting Information, for the storage stability based on PBBD‐Cl‐*β*:BTP‐eC9, *V*_oc_ exhibited almost no decrease (from 0.87 to 0.86 V). Meantime, the *J*_sc_ and FF displayed a slight decrease after storage in a glove box for 216 h as summarized in Table S1, Supporting Information. In order to further explore the slight decline of *J*_sc_ and FF, the absorption spectra of the corresponding PBBD‐Cl‐*β*:BTP‐eC9 blend film (Figure S10, Supporting Information) stored as the device storage‐stability test was measured. As exhibited in Figure S10, Supporting Information, the absorption in the region of 700 to 900 nm slightly decreased, which should be attributed to the degradation and minor changes of the photovoltaic layer, leading to the small decrease of photovoltaic performance. As a result, after being stored for 216 h, the PBBD‐Cl‐*β*:BTP‐eC9‐based device still maintained a PCE of 14.95%, only a 7.8% drop in the initial efficiency. The photostability of the PBBD‐Cl‐*β*:BTP‐eC9‐based device was measured under light soaking conditions and is shown in Figure S8 and Table S2, Supporting Information. The PBBD‐Cl‐*β*:BTP‐eC9‐based device still achieved a high PCE of 13.04% after continuous illumination for 216 h, maintaining 81% of the initial PCE. According to the previous report,^[^ ^11^, ^48^ ^]^ PBBD‐Cl‐*β* can form the non‐covalent Cl···S and Cl···*π* interactions because of the empty 3d orbitals of Cl, which could enhance the intermolecular aggregation and *π*—*π* stacking effect of polymer, leading the tighter and more ordered molecular arrangement for polymer PBBD‐Cl‐*β*. As a result, PBBD‐Cl‐*β*:BTP‐eC9 blend film achieved a solid and cluster‐like phase separation morphology, leading to a fair device stability for PBBD‐Cl‐*β*:BTP‐eC9‐based device. The storage and photostability tests indicated that the chlorinated polymer donor PBBD‐Cl‐*β* showed fair device stability

In summary, we have synthesized two chlorinated D–A conjugated polymers, PBBD‐Cl‐*α* and PBBD‐Cl‐*β*, based on two new building blocks (TTO‐Cl‐*α* and TTO‐Cl‐*β*) respectively formed by introducing the chlorine atom into *α* or *β* position of the upper thiophene unit of benzo[1,2‐b:4,5‐c**′**]dithiophene‐4,8‐dione. The single‐crystal analysis demonstrates that the chlorine‐free TTO showed the *π*—*π* stacking distance (*d*_*π*—*π*_) to be 3.55 Å, and the H···S separation (*d*_H—S_) is 5.93 Å. When H atom at *α* position of thiophene of TTO was replaced by Cl, for TTO‐Cl‐*α*, both *π*—*π* stacking distance (*d*_*π*—*π*_ = 3.48 Å) and Cl···S distance (*d*_Cl—S_ = 4.4 Å) were simultaneously reduced for TTO‐Cl‐*α* compared with that of TTO. And then TTO‐Cl‐*β* showed that the Cl···S non‐covalent interaction can shorten the intermolecular *π*—*π* stacking separation to 3.23 Å, which is much smaller than that of TTO‐Cl‐*α* and TTO. The chlorinated polymer, both PBBD‐Cl‐*α* and PBBD‐Cl‐*β* exhibit deeper HOMO level, and much stronger optical absorption and molecular aggregation than PBBD. After being blended with BTP‐eC9, the PBBD‐Cl‐*β*:BTP‐eC9 blend film displayed high and well‐balanced charge carrier mobilities, and good phase‐separation morphology, improved charge transport and suppressed charge recombination when compared with non‐chlorinated PBBD:BTP‐eC9 blend film. Accordingly, the PBBD‐Cl‐*β*:BTP‐eC9‐based PSCs with three improved key factors achieved an outstanding PCE of 16.20%, much higher than PBBD:BTP‐eC9 (10.06%) and PBBD‐Cl‐*α*:BTP‐eC9 (13.35%) based devices. Therefore, this demonstrates the reasonable positioning of chlorine in a donor polymer plays a significant role in molecular arrangement and also can provide new insight into the rational design of a chlorination strategy for future polymer solar cell applications.

## Conflict of Interest

The authors declare no conflict of interest.

## Supporting information

Supporting Information
