# Supplementary material for: Chlorinated Benzo[1,2‐b:4,5‐c′]dithiophene‐4,8‐dione Polymer Donor: A Small Atom Makes a Big Difference
Source: Adv Sci (Weinh). 2021 Jan 4;8(4):2003641. doi: 10.1002/advs.202003641 (PMC7887605; doi:10.1002/advs.202003641)
Supplement: Supplementary file 1 — Supporting Information [file ADVS-8-2003641-s001.pdf]

WILEY-VCH

© 2020 Wiley-VCH GmbH

ADVANCED SCIENCE
Open Access

Supporting Information

for *Adv. Sci.*, DOI: 10.1002/advs.202003641

Chlorinated Benzo[1,2-b:4,5-c']dithiophene-4,8-dione Polymer Donor: A Small Atom Makes A Big Difference

*Pengjie Chao, Hui Chen, Mingrui Pu, Yulin Zhu, Liang Han, Nan Zheng, Jiadong Zhou, Xiaoyong Chang, Daize Mo, Zengqi Xie, Hong Meng, and Feng He**

Supporting Information

Chlorinated Benzo[1,2-b:4,5-c']dithiophene-4,8-dione Polymer Donor: A Small Atom Makes A Big Difference

*Pengjie Chao, Hui Chen, Mingrui Pu, Yulin Zhu, Liang Han, Nan Zheng, Jiadong Zhou, Xiaoyong Chang, Daize Mo, Zengqi Xie, Hong Meng, and Feng He**

Dr. P.J. Chao, Dr. H. Chen, Y.L. Zhu, M.R. Pu, Dr. D.Z. Mo, Dr. L. Han, Dr. X.Y. Chang and Prof. F. He
Shenzhen Grubbs Institute and Department of Chemistry, Southern University of Science and Technology, Shenzhen 518055, China
E-mail: hef@sustech.edu.cn

Dr. P. J. Chao and Prof. H. Meng
School of Advanced Materials, Peking University Shenzhen Graduate School, Peking University, Shenzhen, 518055, China

Dr. H. Chen
Academy for Advanced Interdisciplinary Studies and Department of chemistry, Southern University of Science and Technology, Shenzhen 518055, China

Dr. N. Zheng, Dr. J.D. Zhou, Prof. Z.Q. Xie
Institute of Polymer Optoelectronic Materials and Devices, State Key Laboratory of Luminescent Materials and Devices, South China University of Technology, Guangzhou 510640, China

Prof. F. He
Guangdong Provincial Key Laboratory of Catalysis, Southern University of Science and Technology, Shenzhen 518055, China

1. Experimental Section

Measurements: ^{1}H NMR and ^{13}C NMR spectra were recorded on Bruker Avance-400/500 spectrometers. Mass spectra (high resolution mass spectrometer (HRMS)) were determined on an Autoflex III matrix-assisted laser desorption ionization mass spectrometer (MALDI-TOF-MS). Gel permeation chromatography (GPC) was performed on Agilent Technologies 1260 infinity Ⅱhigh temperature GPC system using 1,2,4-trichlorobenzene (TCB) as eluent at 150 ^{o}C. Solution and thin film optical absorption spectra were measured with a UV-Vis spectrophotometer (Shimadzu, UV3600). The thin films of the polymers were spin-coated from their solutions in chloroform, and then the film absorption spectra were measured. The electrochemical cyclic voltammetry (CV) was carried out on a CHI 660E Electrochemicacl Workstation (Shanghai Chenhua Instrumental Co., Ltd. China), with glassy carbon disk, Pt wire and Ag/Ag+ electrode as working electrode, counter electrode and reference electrode in an acetonitrile solution of 0.1 mol L^{-1} Tetrabutylammonium phosphorus hexafluoride (n-Bu_4NPF_6) at a potential scan rate of 100 mV s^{-1} under a argon atmosphere. Tapping mode atom force microscopy (TM-AFM) images were taken on a NanoScope IIIa controller (Veeco Metrology Group/Digital Instruments, Sant a Barbara, CA), using built-in software (version V6.13R1) to capture images. Transmission electron microscopy (TEM) images were acquired using a HITACHI H-7650 electron microscope operating at an acceleration voltage of 100 kV. The thickness of the blend films was determined by a Dektak 6 M surface profilometer. All J–V curves were captured under an AAA solar simulator

(SAN-EI) calibrated by a standard single-crystal Si photovoltaic cell (certificated by National Institute of Metrology).

2. Device Fabrication and Testing

The device structure was ITO/PEDOT:PSS/polymer:BTP-eC9/PNDIT-F3N/Ag. ITO-coated glass substrates were cleaned with deionized water, acetone and isopropyl alcohol for 30 minutes once time and dried in the drying oven at 80 ^{o}C for 12 h before used. A PEDOT:PSS interlayer from a precursor solution was spin-coated onto the pre-cleaned and UV-treated ITO substrates, then heated at 150 ^{o}C for 10 min. The ITO glass was then placed in the UV-ozone for 15 minutes and the sol-gel-derived ZnO films was spin-coated onto the ITO sustrated followed by thermal treatment at 200 °C for 30 min and cooled to room temperature under vacuum. The mixture of polymer/BTP-eC9 (1:1.2 by wt/wt ratio) was dissolved in chlorobenzene to obtain 10 mg mL^{-1} of solution. The blend was stirred in the glove box for overnight. The active layer was spin-coating at 2000 rpm for 60 s to get the blend film. A 100 nm Ag layer were subsequently evaporated through a shadow mask to define the active area of the devices. The power conversion efficiencies (PCEs) were tested under AM 1.5G irradiation with the intensity of 100 mW cm^{-2} (Enlitech.Inc) which was calibrated by a NREL certified standard silicon cell (4 cm^{-2}). The *J-V* curves were recorded with the computer-controlled Keithley 2400 sourcemeter in a dry box under an inert atmosphere. The external quantum efficiency (EQE) spectra were measured through the measurement of solar cell spectral response measurement system QE-R3011 (Enli Technology Ltd., Taiwan).

The mobility of electrons was tested by fitting the current-bias characteristics in the dark utilizing a field-independent space charge limited current (SCLC) model following the Mott-Gurney law $J = \frac{9}{8}\varepsilon_0\varepsilon_r\mu\frac{V^2}{L^3}$. The structures of hole-only and electron-only devices are ITO/PEDOT:PSS/polymer:BTP-eC9/MoO_3/Ag and ITO/ZnO/polymer:BTP-eC9/PNDIT-F3N/Al, respectively. The processing conditions used for the active layers were the optimized ones. Charge mobility was extracted by

fitting the current density–voltage curves, recorded under dark conditions, with the Mott-Gurney equation. The mobility was determined by fitting the dark current to the model of a single carrier SCLC, which is described by the equation

$$J = \frac{9}{8}\varepsilon_0 \varepsilon_r \mu_h \frac{V^2}{d^3}$$

where J is the current, μ_h is the zero-field mobility, ε_0 is the permittivity of free space, ε_r is the relative permittivity of the material, d is the thickness of the active layer, and V is the effective voltage. The effective voltage can be obtained by subtracting the built-in voltage (V_{bi}) and the voltage drop (V_s) from the substrate's series resistance from the applied voltage (V_{appl}), $V = V_{appl} - V_{bi} - V_{s.}$ The photo-ability of device was determined by the long-time stable LED white light soaking test system (Enlitech.Inc). The light source area is 10 cm*10 cm with favorable uniformity. The light intensity was outputted in 100 mW cm^{-2} by control system. The operating temperature of setup is about 50 °C, the humidity is 10% in glove box.

3. Materials

All chemicals and solvents were reagent grades and purchased from Aldrich, Energy, Derthon and solarmer, respectively. All starting reagents were obtained commercially as analytical grade and used directly without any purification unless stated otherwise. Toluene and THF were distilled over sodium/benzophenone and calcium hydride under N_2 prior to use.

4. Synthesis and characterization

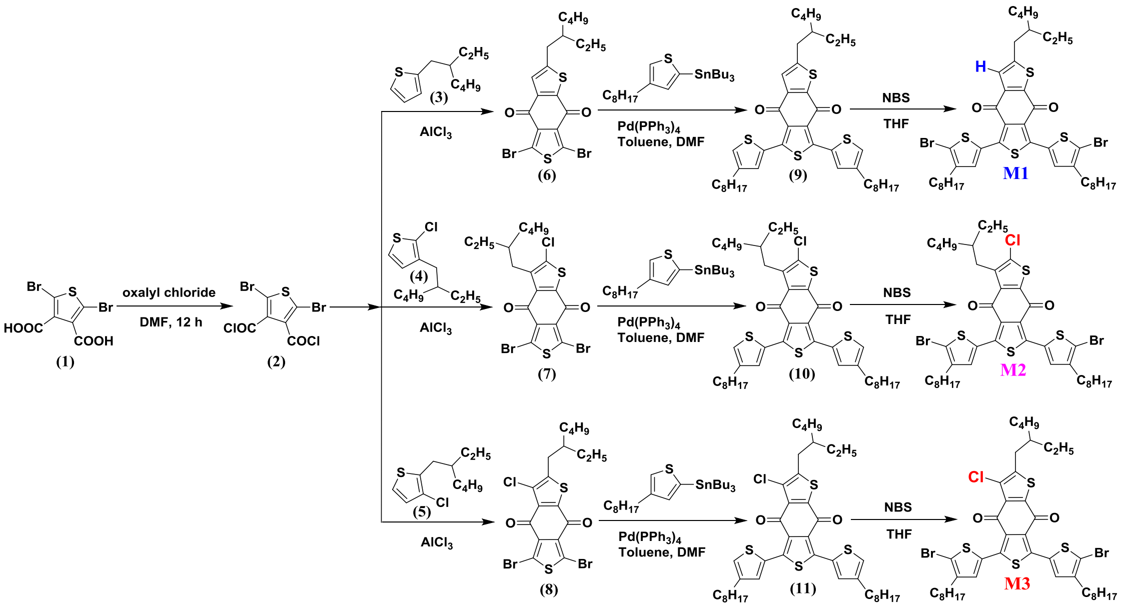

Scheme S1. Synthetic routes of compound M1, M2 and M3.

2-(2-ethylhexyl)thiophene (3). Under protection of argon, *n*-BuLi (148.57 ml, 2.4M, 356.6 mmol) was slowly added to thiophene (30.00 g, 50.93 mmol) in THF (400 ml) at -78 °C, and the mixture was kept at -78 °C for 5h. Then 2-ethylhexyl bromide (65.42 g, 33.88 mmol) was added, and then the mixture stirred for 16h at 60 °C. After cooling down to the room temperature, the mixture was extracted by ethyl acetate. The organic phase was dried over anhydrous sodium sulfate and concentrated by a rotary evaporator. Further purification was carried out by reduced pressure distillation to obtain the production as a colorless oil (30.59 g, 70.01%). ^{1}H NMR (500 MHz, Chloroform-*d*) δ 7.11 (d, *J* = 5.1 Hz, 1H), 6.95 – 6.89 (m, 1H), 6.76 (d, *J* = 3.1 Hz, 1H), 2.76 (d, *J* = 6.8 Hz, 2H), 1.61 – 1.55 (m, 1H), 1.45 – 1.20 (m, 8H), 0.88 (t, *J* = 7.2 Hz, 6H). ^{13}C NMR (126 MHz, Chloroform-*d*) δ 144.37, 126.53, 124.94, 122.90,

41.46, 33.81, 32.32, 28.84, 25.47, 23.00, 14.13, 10.81.

2-Chloro-3-(2-ethylhexyl)thiophene (4). 3-(2-ethylhexyl)thiophene (23.35 g, 118.90 mmol) was added into AcOH/$CHCl_3$ (200/200 mL). Then N-chlorosuccinimide (NCS) (12.24 g, 91.67 mmol) was added in one portion. The reaction mixture was stirred at room temperature for 4 h, water was added into the mixture, the mixture was extracted with ethyl acetate , and the organic layer was washed with brine and dried over anhydrous magnesium sulfate. The solvent was removed at a reduced pressure, the residue was purified by column chromatography on silica gel with petroleum ether to give a colorless oil (25.62 g, 93.37%). ^{1}H NMR (400 MHz, Chloroform-*d*) δ 7.02 (d, *J* = 5.7 Hz, 1H), 6.76 (d, *J* = 5.7 Hz, 1H), 2.50 (d, *J* = 7.1 Hz, 2H), 1.59 (dd, *J* = 12.5, 6.4 Hz, 1H), 1.39 – 1.17 (m, 8H), 0.88 (t, *J* = 7.3 Hz, 6H). ^{13}C NMR (101 MHz, Chloroform-*d*) δ 138.37, 128.45, 124.95, 121.80, 39.93, 32.47, 32.14, 28.78, 25.66, 23.03, 14.12, 10.79. GC-MS (M^+): calculated for $C_{18}H_{18}Br_2O_2S_2$ [M+], 230.7940; found: 230.1.

3-Chloro-2-(2-ethylhexyl)thiophene (5). 3-chlorothiophene (20.00 g, 168.70 mmol) was added dropwise to a solution of lithium diisopropylamide(88.6 ml, 177.10 mmol) at 0 ^{o}C. After stirring the mixture for 2 h, 2-ethylhexyl bromide (33.2 mL, 185.50 mmol) was added to the mixture at 0 ℃.After the addition, the mixture was heated at 60 ^{o}C for 16 h . The reaction mixture was poured into the water, extracted with ethyl acetate. The organic layer was dried over magnesium sulfate and the solvent was removed via rotary evaporation, and the residue was purified through vacuum distillation to give a colorless liquid (33.15 g, yield = 86.36%). ^{1}H NMR (400 MHz,

$CDCl_3$) δ 7.10 (d, *J* = 5.4 Hz, 1H), 6.85 (d, *J* = 5.4 Hz, 1H), 2.72 (d, *J* = 7.0 Hz, 2H), 1.69 – 1.57 (m, 1H), 1.40 – 1.22 (m, 8H), 0.97 – 0.82 (m, 6H). ^{13}C NMR (126 MHz, $CDCl_3$) δ 136.93, 127.37, 122.59, 122.02, 40.91, 32.37, 31.70, 28.75, 25.59, 22.99, 14.09, 10.78. HRMS: calculated for $C_{12}H_{19}ClS$ [M^+]: 230.7940; Found: 231.0969.

5,7-dibromo-2-(2-ethylhexyl)benzo[1,2-b:4,5-c']dithiophene-4,8-dione (6) Oxalyl chloride (2.1 mL) was slowly added to 2,5-dibromothiophene-3,4-dicarboxylic acid (1) (1 g, 3.03 mmol) and DMF (1 drop) in dry dichloromethane (DCM) (20 mL). The mixture was stirred for 12 h at room temperature. The solvent was removed under vacuum to obtain crude 2,5-dibromothiophene-3,4-dicarbonyl dichloride, which was used for next step without further purification. To a stirred solution of the dicarbonyl dichloride (2) (1.5 g, 4.09 mmol) and 2-(2-ethylhexyl)thiophene (3) (0.80 g, 4.09 mmol) in dry 1,2-dichloroethane, $AlCl_3$ (2.18 g, 16.36 mmol) was added in small portions at 0 ° C. The mixture was allowed to stir at 0 ° C for 30 min and then at room temperature for 6 h. The mixture was poured into ice with 1 mol/L hydrochloric acid and then extracted with DCM . The organic layer was collected and the volatile solvent was removed under vacuum. The crude product was purified through a silica gel column with petroleum ether/dichloromethane (5:1 by volume) to give a purple solid (0.72 g, 36%). ^{1}H NMR (400 MHz, Chloroform-*d*) δ 7.33 (s, 1H), 2.83 (d, *J* = 6.9 Hz, 2H), 1.66 (m, 1H), 1.42 – 1.23 (m, 8H), 0.91 (t, *J* = 7.4 Hz, 6H). ^{13}C NMR (101 MHz, Chloroform-*d*) δ 173.87, 172.17, 158.26, 144.97, 144.27, 133.45, 133.26, 124.64, 120.90, 120.49, 41.46, 34.88, 32.31, 28.75, 25.53, 22.90, 14.08, 10.76. HRMS ($MALDI^+$): calculated for $C_{18}H_{18}Br_2O_2S_2$ [M+], 490.2680; found: 489.8924.

5,7-dibromo-2-chloro-3-(2-ethylhexyl)benzo[1,2-b:4,5-c']dithiophene-4,8-dione (7)

Oxalyl chloride (66.38 g) was slowly added to 2,5-dibromothiophene-3,4-dicarboxylic acid (1) (21.57 g, 65.37 mmol) and DMF (1 drop) in dry dichloromethane (DCM) (250 mL). The mixture was stirred for 12 h at room temperature. The solvent was removed under vacuum to obtain crude 2,5-dibromothiophene-3,4-dicarbonyl dichloride, which was used for next step without further purification. To a stirred solution of the dicarbonyl dichloride (2) (12 g, 31.35 mmol) and 2-chloro-5-(2-ethylhexyl)thiophene (3) (7.24 g, 31.35 mmol) in dry 1,2-dichloroethane, $AlCl_3$ (16.72 g, 125.4 mmol) was added in small portions at 0 ° C. The mixture was allowed to stir at 0 ° C for 30 min and then at room temperature for 6 h. The mixture was poured into ice with 1 mol/L hydrochloric acid and then extracted with DCM . The organic layer was collected and the volatile solvent was removed under vacuum. The crude product was purified through a silica gel column with petroleum ether/dichloromethane (5:1 by volume) to give a light yellow solid (9.94 g, 60.43%). ^{1}H NMR (400 MHz, Chloroform-*d*) δ 2.96 (d, *J* = 7.3 Hz, 1H), 1.69 (m, 1H), 1.45 – 1.15 (m, 8H), 0.89 (m, 6H). ^{13}C NMR (101 MHz, Chloroform-*d*) δ 173.73, 171.67, 145.17, 142.03, 139.98, 137.84, 133.23, 132.22, 121.34, 120.82, 39.38, 32.32, 31.79, 28.52, 25.60, 23.11, 14.13, 10.85. HRMS (MALDI^{+}): calculated for $C_{18}H_{17}Br_2ClO_2S_2$ [M+], 524.7100; found: 525.8569.

5,7-dibromo-3-chloro-2-(2-ethylhexyl)benzo[1,2-b:4,5-c']dithiophene-4,8-dione (8)

Oxalyl chloride (2.1 mL) was slowly added to 2,5-dibromothiophene-3,4-dicarboxylic acid (1) (1 g, 3.03 mmol) and DMF (1 drop) in dry dichloromethane (DCM) (20 mL).

The mixture was stirred for 12 h at room temperature. The solvent was removed under vacuum to obtain crude 2,5-dibromothiophene-3,4-dicarbonyl dichloride, which was used for next step without further purification. To a stirred solution of the dicarbonyl dichloride (2) (1.76 g, 4.79 mmol) and 3-chloro-2-(2-ethylhexyl)thiophene (4) (1.11 g, 4.79 mmol) in dry 1,2-dichloroethane, $AlCl_3$ (2.55 g, 19.16 mmol) was added in small portions at 0 ° C. The mixture was allowed to stir at 0 ° C for 30 min and then at room temperature for 6 h. The mixture was poured into ice with 1 mol/L hydrochloric acid and then extracted with DCM . The organic layer was collected and the volatile solvent was removed under vacuum. The crude product was purified through a silica gel column with petroleum ether/dichloromethane (5:1 by volume) to give a light yellow solid (1.08 g, 43.03%). ^{1}H NMR (400 MHz, Chloroform-*d*) δ 2.86 (d, *J* = 7.1 Hz, 2H), 1.74 (m, 1H), 1.48 – 1.19 (m, 8H), 0.91 (m, 6H). ^{13}C NMR (101 MHz, Chloroform-*d*) δ 172.56, 171.30, 150.34, 144.08, 137.38, 133.34, 132.33, 124.20, 121.55, 120.95, 40.69, 32.67, 32.39, 28.65, 25.66, 22.92, 14.08, 10.73. HRMS ($MALDI^+$): calculated for $C_{18}H_{17}Br_2ClO_2S_2$ [M+], 524.7100; found: 523.8317.

2-(2-ethylhexyl)-5,7-bis(4-octylthiophen-2-yl)benzo[1,2-b:4,5-c']dithiophene-4,8-dione (9) $Pd(PPh_3)_4$ (177 mg) was added to a solution of compound 5 (1.50 g, 2.86 mmol) and tributyl(4-octylthiophen-2-yl)stannane (4.46 g, 8.57 mmol) in 100 mL of dry toluene. The mixture was refluxed in an argon atmosphere for 24 h. After the removal of the solvent at a reduced pressure, the residue was purified by column chromatography on a silica gel column with petroleum ether/dichloromethane (5:1 by volume) to give a red solid (1.92 g, 86.88%). ^{1}H NMR (400 MHz, Chloroform-*d*) δ

7.75 – 7.69 (m, 2H), 7.33 (s, 1H), 7.14 (d, *J* = 2.7 Hz, 2H), 2.83 (d, *J* = 6.7 Hz, 2H), 2.65 (t, *J* = 7.3 Hz, 4H), 1.75 – 1.61 (m, 5H), 1.46 – 1.19 (m, 28H), 0.90 (m, 12H). ^{13}C NMR (101 MHz, Chloroform-*d*) δ 176.03, 174.42, 155.22, 145.56, 144.37, 144.33, 144.02, 143.82, 143.74, 132.56, 132.49, 132.43, 130.05, 129.66, 125.18, 124.50, 41.41, 34.79, 32.38, 31.92, 30.51, 30.42, 29.46, 29.38, 29.31, 28.84, 25.54, 22.95, 22.71, 14.15, 14.13, 10.81. HRMS (MALDI+): calculated for $C_{42}H_{56}O_2S_4$ [M+], 721.1480; found: 721.4336.

2-chloro-3-(2-ethylhexyl)-5,7-bis(4-octylthiophen-2-yl)benzo[1,2-b:4,5-c']dithiophene-4,8-dione (10) $Pd(PPh_3)_4$ (165.30 mg) was added to a solution of compound 6 (1.50 g, 2.86 mmol) and tributyl(4-octylthiophen-2-yl)stannane (4.16 g, 8.57 mmol) in 100 mL of dry toluene. The mixture was refluxed in an argon atmosphere for 24 h. After the removal of the solvent at a reduced pressure, the residue was purified by column chromatography on a silica gel column with petroleum ether/dichloromethane (5:1 by volume) to give a red solid (1.50 g, 69.44%). ^{1}H NMR (400 MHz, Chloroform-*d*) δ 7.72 (d, *J* = 19.7 Hz, 2H), 7.14 (s, 2H), 2.97 (d, *J* = 7.1 Hz, 2H), 2.65 (t, *J* = 7.6 Hz, 4H), 1.82 – 1.58 (m, 5H), 1.42 – 1.24 (m, 28H), 0.90 (m, 12H). ^{13}C NMR (101 MHz, Chloroform-*d*) δ 175.79, 173.70, 145.77, 144.32, 144.13, 143.94, 143.67, 141.54, 139.89, 136.44, 132.62, 132.55, 132.44, 132.33, 129.88, 128.32, 125.42, 125.35, 39.68, 32.68, 31.93, 31.68, 30.51, 30.49, 30.42, 29.47, 29.41, 29.32, 28.77, 25.84, 23.18, 22.72, 14.21, 14.16, 10.99. HRMS (MALDI+): calculated for $C_{42}H_{55}ClO_2S_4$ [M+], 755.5900; found: 755.4385.

3-chloro-2-(2-ethylhexyl)-5,7-bis(4-octylthiophen-2-yl)benzo[1,2-b:4,5-c']dithio-phene-4,8-dione (11) $Pd(PPh_3)_4$ (165 mg) was added to a solution of compound 6 (1.50 g, 2.86 mmol) and tributyl(4-octylthiophen-2-yl)stannane (4.16 g, 8.57 mmol) in 100 mL of dry toluene. The mixture was refluxed in an argon atmosphere for 24 h. After the removal of the solvent at a reduced pressure, the residue was purified by column chromatography on a silica gel column with petroleum ether/dichloromethane (5:1 by volume) to give a red solid (1.85 g, 85.65%). ^{1}H NMR (400 MHz, Chloroform-*d*) δ 7.72 (d, *J* = 30.1 Hz, 2H), 7.15 (s, 2H), 2.86 (d, *J* = 7.1 Hz, 2H), 2.65 (t, *J* = 8.3 Hz, 4H), 1.79 – 1.71 (m, 1H), 1.66 (m, 4H), 1.47 – 1.16 (m, 28H), 1.00 – 0.78 (m, 12H). ^{13}C NMR (101 MHz, Chloroform-*d*) δ 174.76, 173.47, 148.84, 144.84, 144.58, 144.40, 143.95, 143.72, 137.43, 132.68, 132.59, 132.29, 132.23, 130.17, 128.46, 125.47, 125.39, 123.65, 40.65, 32.56, 32.41, 31.90, 30.48, 30.39, 29.45, 29.37, 29.29, 28.69, 25.67, 22.95, 22.69, 14.13, 14.10, 10.75. HRMS ($MALDI^+$): calculated for $C_{42}H_{55}ClO_2S_4$ [M+], 755.5900; found: 756.3906.

5,7-bis(5-bromo-4-octylthiophen-2-yl)-2-(2-ethylhexyl)benzo[1,2-b:4,5-c']dithiop-hene-4,8-dione (M1) Compound 7 (1.92 g, 2.66 mmol) was added into THF (30 mL). After the solid dissolved completely, N-bromosuccinimide (NBS) (1.14 g, 6.39 mmol) was added in one portion. The reaction mixture was stirred at room temperature for 4 h, water was added into the mixture, the mixture was extracted with ethyl acetate , and the organic layer was washed with brine and dried over anhydrous magnesium sulfate. The solvent was removed at a reduced pressure, the residue was purified by column chromatography on silica gel with petroleum ether to give a red sticky solid (2.16 g,

92.31%). ^{1}H NMR (400 MHz, Chloroform-*d*) δ 7.39 (d, *J* = 3.8 Hz, 2H), 7.21 (d, *J* = 5.6 Hz, 1H), 2.78 (d, *J* = 4.2 Hz, 2H), 2.54 (s, 4H), 1.61 (s, 5H), 1.32 (m, 28H), 0.89 (m, 12H). ^{13}C NMR (101 MHz, Chloroform-*d*) δ 175.64, 174.00, 155.71, 155.69, 145.02, 143.94, 143.11, 142.74, 142.36, 132.18, 132.15, 131.40, 129.26, 129.22, 128.89, 128.85, 124.40, 116.68, 41.32, 34.79, 32.36, 31.91, 29.76, 29.49, 29.40, 29.30, 25.51, 22.95, 22.70, 14.14, 10.79. HRMS (MALDI+): calculated for $C_{42}H_{54}Br_2O_2S_4$ [M+], 878.9400; found: 878.3011.

5,7-bis(5-bromo-4-octylthiophen-2-yl)-2-chloro-3-(2-ethylhexyl)benzo[1,2-b:4,5-c']dithiophene-4,8-dione (M2) Compound 8 (1.40 g, 1.85 mmol) was added into THF (80 mL). After the solid dissolved completely, N-bromosuccinimide (NBS) (791.45 mg, 4.45 mmol) was added in one portion. The reaction mixture was stirred at room temperature for 4 h, water was added into the mixture, the mixture was extracted with ethyl acetate , and the organic layer was washed with brine and dried over anhydrous magnesium sulfate. The solvent was removed at a reduced pressure, the residue was purified by column chromatography on silica gel with petroleum ether to give a red sticky solid (1.54 g, 90.82%). ^{1}H NMR (400 MHz, Chloroform-*d*) δ 7.44 (d, *J* = 7.5 Hz, 2H), 2.93 (ddd, *J* = 45.9, 13.1, 7.1 Hz, 2H), 2.65 – 2.50 (m, 4H), 1.70 – 1.58 (m, 5H), 1.41 – 1.25 (m, 28H), 1.07 – 0.82 (m, 12H). ^{13}C NMR (101 MHz, Chloroform-*d*) δ 175.37, 173.20, 145.16, 143.20, 142.77, 142.51, 142.27, 141.54, 139.41, 136.97, 132.12, 131.92, 131.42, 131.28, 128.94, 127.44, 127.43, 117.14, 39.72, 32.87, 31.91, 31.64, 29.76, 29.73, 29.52, 29.50, 29.42, 29.32, 28.97, 25.88, 23.25, 22.71, 14.38, 14.15, 11.01. HRMS (MALDI+): calculated for $C_{42}H_{53}Br_2ClO_2S_4$ [M^+], 913.3820;

found: 913.2681.

5,7-bis(5-bromo-4-octylthiophen-2-yl)-3-chloro-2-(2-ethylhexyl)benzo[1,2-b:4,5-c']dithiophene-4,8-dione (M3) Compound 8 (1.85 g, 2.45 mmol) was added into THF (30 mL). After the solid dissolved completely, N-bromosuccinimide (NBS) (1.04 g, 5.87 mmol) was added in one portion. The reaction mixture was stirred at room temperature for 4 h, water was added into the mixture, the mixture was extracted with ethyl acetate , and the organic layer was washed with brine and dried over anhydrous magnesium sulfate. The solvent was removed at a reduced pressure, the residue was purified by column chromatography on silica gel with petroleum ether to give a red sticky solid (2.19 g, 97.77%). ^{1}H NMR (400 MHz, Chloroform-*d*) δ 7.36 (d, *J* = 4.4 Hz, 1H), 7.32 (d, *J* = 3.9 Hz, 1H), 2.76 (d, *J* = 6.9 Hz, 2H), 2.52 (q, *J* = 6.0, 5.2 Hz, 4H), 1.76 – 1.66 (m, 1H), 1.59 (s, 4H), 1.32 (m, 28H), 0.90 (m, 12H). ^{13}C NMR (101 MHz, Chloroform-*d*) δ 174.36, 172.89, 149.32, 144.24, 143.38, 142.94, 142.52, 142.36, 136.88, 131.97, 131.90, 131.49, 131.40, 129.16, 127.61, 127.56, 123.74, 117.15, 40.57, 32.56, 32.41, 31.91, 29.74, 29.48, 29.41, 29.32, 28.68, 25.66, 22.98, 22.71, 10.74. HRMS (MALDI+): calculated for $C_{42}H_{53}Br_2ClO_2S_4$ [M+], 913.3820; found: 913.2688.

Polymerization of PBBD. To a 25 mL flask, compound M1 (186.90 mg, 0.2126 mmol), compound M3 (200 mg, 0.2126 mmol) and $Pd(PPh_3)_4$ (9.83 mg, 0.0085 mmol) were added under argon, then the reaction container was purged with argon for 20 min to remove O_2. After the addition of toluene (8.5 mL), the reactant mixture was heated to reflux and maintained at the same temperature for 18 h. After cooling to room

temperature, the mixture was poured into methanol (200 ml), then filtered through a Soxhlet thimble, which was then subjected to Soxhlet extraction with methanol, acetone, hexane and chloroform. The polymer was recovered as solid from the chloroform fraction by precipitation from methanol. The solid was dried under vacuum. Yield: 237.40 mg (81.98%). The polymer was thermally stable up to 398 ^{o}C (5% weight loss by TGA). GPC:M_w=66.28 kDa; M_n=30.60 kDa; PDI=2.16. ^{1}H NMR (400 MHz, $CDCl_2CDCl_2$, 100 ^{o}C) δ (ppm) 7.78-7.27 (br, ArH), 2.93-2.45 (br, Ar-CH_2), 1.77-1.72 (br, -CH and –CH_2), 1.52-1.30 (br, -CH_2), 1.02-0.89 (br, -CH_3).

Polymerization of PBBD-Cl-*α*. PBBD-Cl-*α* was prepared using the same procedure as PBBD. Yield: 231.20 mg (77.87%). The polymer was thermally stable up to 400 ^{o}C (5% weight loss by TGA). GPC:M_w=79.43 kDa; M_n=33.52 kDa; PDI=2.36. ^{1}H NMR (400 MHz, $CDCl_2CDCl_2$, 100 ^{o}C) δ (ppm) 7.82-7.25 (br, ArH), 2.96 (br, Ar-CH_2), 1.85 (br, -CH and -CH_2), 1.50 (br, -CH_2), 1.07 (br, -CH_3).

Polymerization of PBBD-Cl-*β*. PBBD-Cl-*β* was prepared using the same procedure as PBBD. Yield: 246.90 mg (83.16%). GPC:M_w=56.90 kDa; M_n=31.07 kDa; PDI=1.83. The polymer was thermally stable up to 404 ^{o}C (5% weight loss by TGA). ^{1}H NMR (400 MHz, $CDCl_2CDCl_2$, 100 ^{o}C) δ (ppm) 7.79-7.28 (br, ArH), 2.87-2.46 (br, Ar-CH_2), 1.77 (br, -CH and -CH_2), 1.49-1.29 (br, -CH_2), 1.02-0.89 (br, -CH_3).

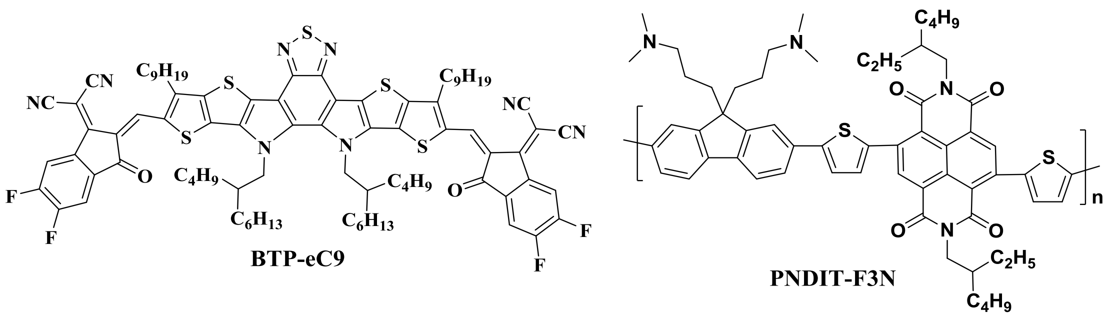

Figure S1. Structure of BTP-eC9 and PNDIT-F3N.

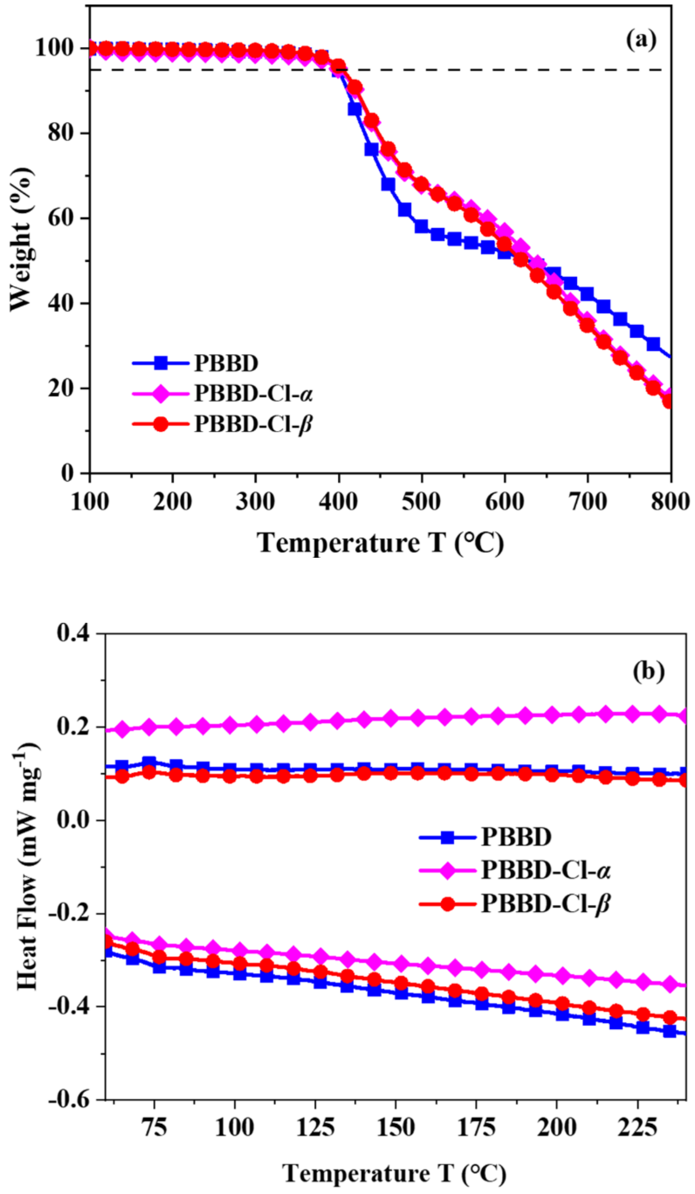

Figure S2. (a) TGA curves and (b) DSC curves of PBBD, PBBD-Cl-*α* and PBDD-Cl-*β*.

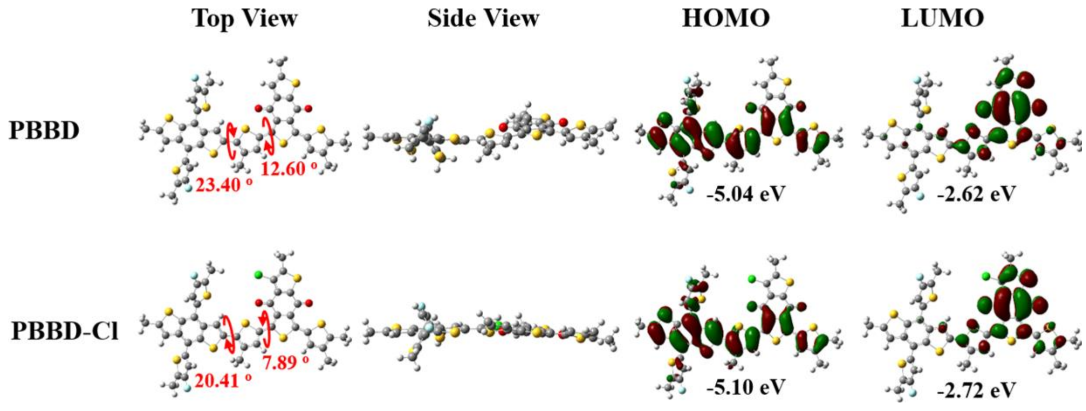

Figure S3. Optimized geometries and frontier orbitals of polymer repeat unit.

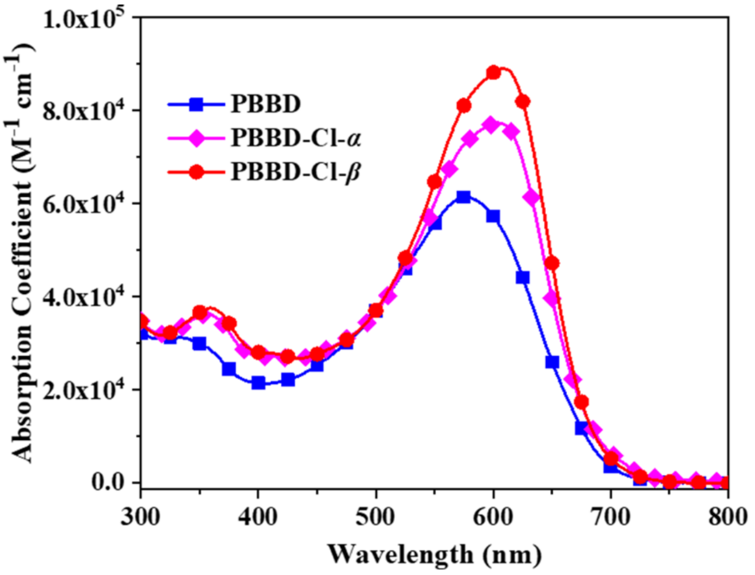

Figure S4. Absorbance coefficient spectra of polymer PBBD, PBBD-Cl-α and PBDD-Cl-β in solution.

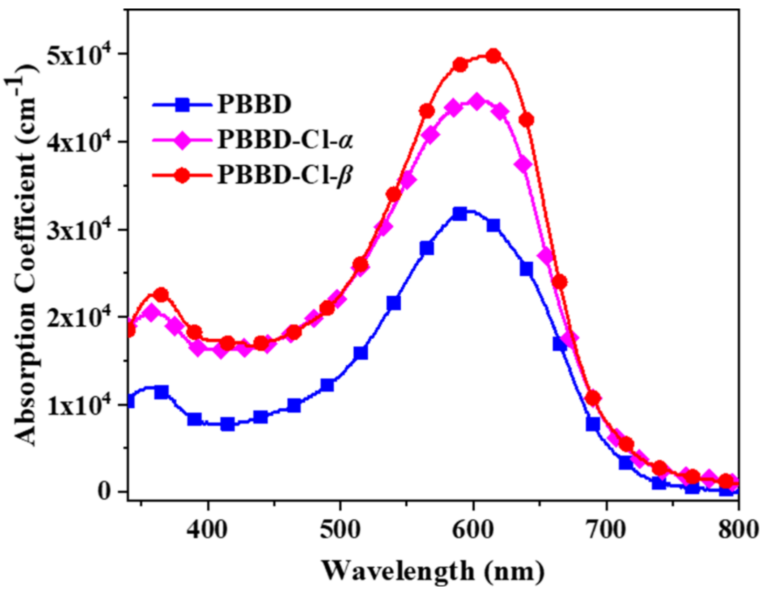

Figure S5. Absorbance coefficient spectra of polymer PBBD, PBBD-Cl-α and PBDD-Cl-β in film.

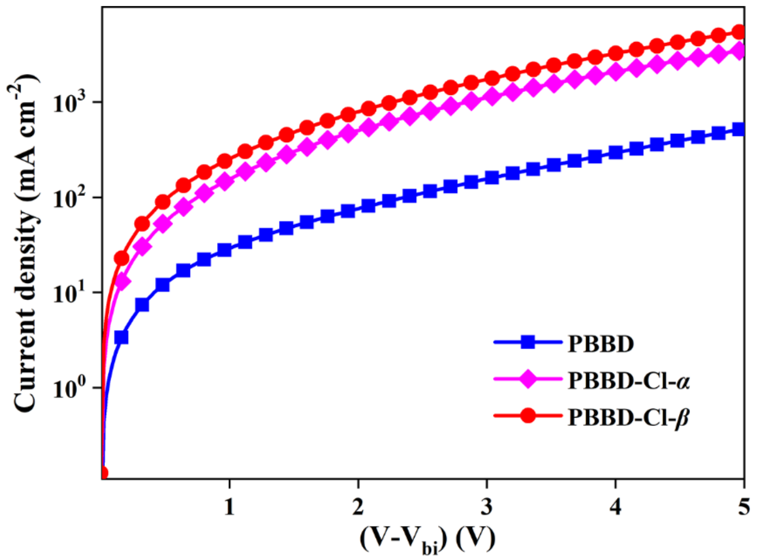

Figure S6. The curves of the hole-only devices based on polymer neat film.

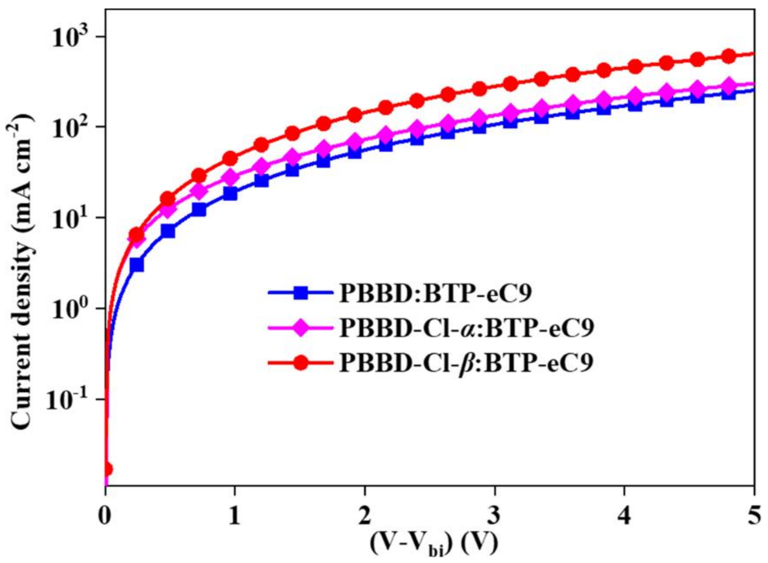

Figure S7. The curves of the hole-only devices based on polymer:BTP-eC9.

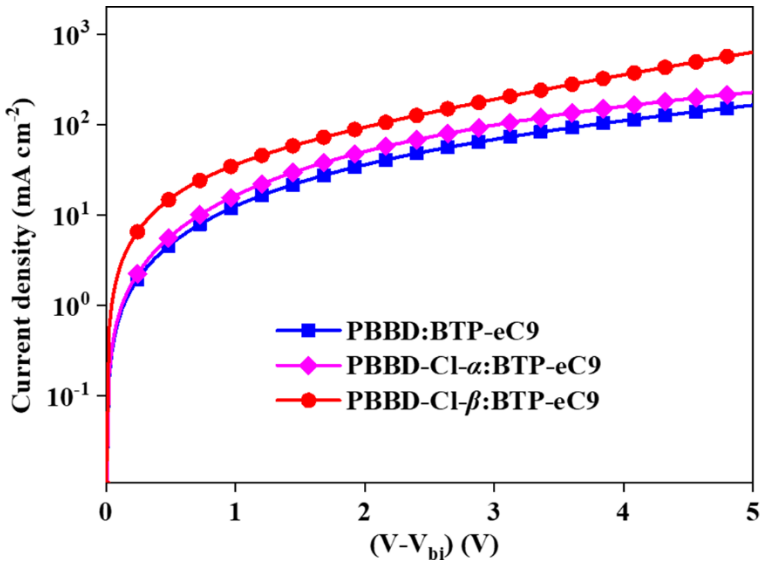

Figure S8. The curves of the electron-only devices based on polymer: BTP-eC9.

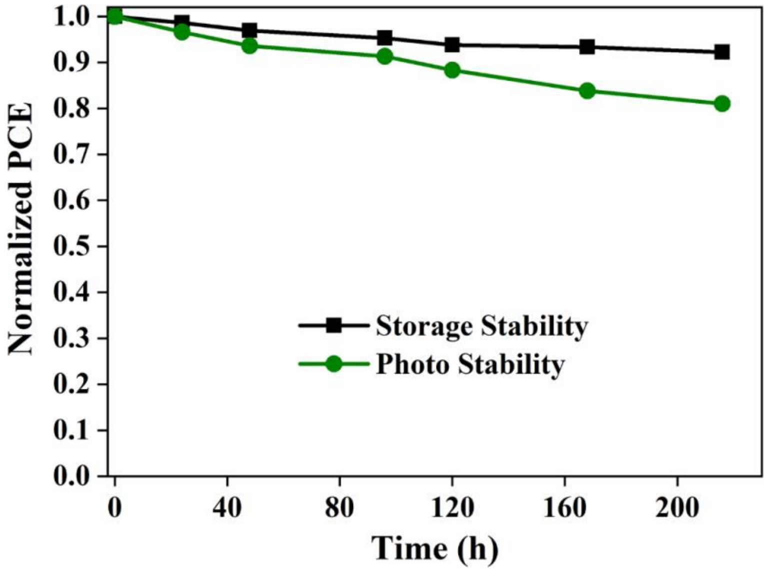

Figure S9. The photovoltaic performance parameters of the storage and photo stability based on optimized PBBD-Cl:BTP-eC9 device.

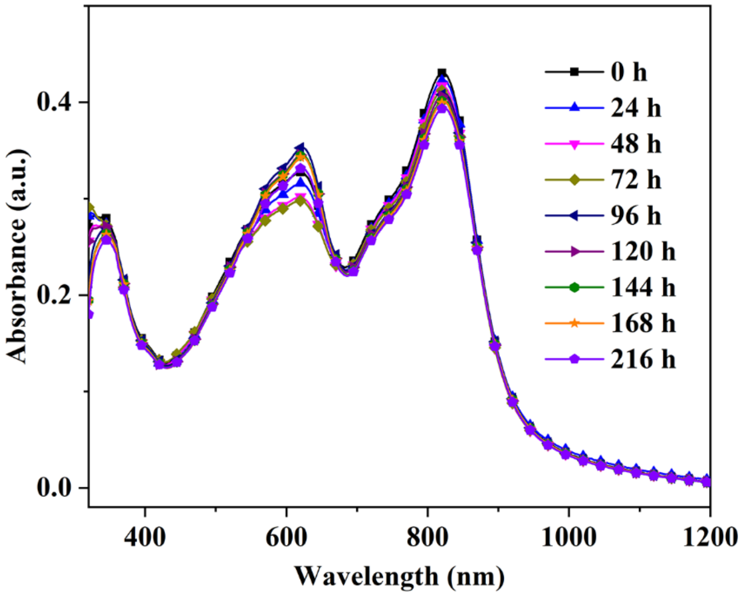

Figure S10. Absorption spectra of blend film of PBBD-Cl-β:BTP-eC9 stored as the device storage-stability test.

Table S1. Photovoltaic performance parameters of the lifetime tests based on PBBD-Cl PSCs stored in glove box under dark.

Time (h)	V_{oc} (V)	J_{sc} (mA cm^{-2})	FF (%)	PCE (%)	Normalized PCE
0 h	0.87	24.59	75.53	16.20	1
24 h	0.87	24.47	74.98	15.98	0.986
48 h	0.87	24.32	74.22	15.71	0.969
96 h	0.87	24.16	73.52	15.44	0.953
120 h	0.86	24.04	73.22	15.21	0.938
168 h	0.86	24.02	72.86	15.12	0.933
216 h	0.86	23.98	72.23	14.95	0.922

Table S2. Photovoltaic performance parameters of the photo-stability tests of optimal PSCs under light soaking condition based on PBTT-F:BTP-eC9 PSCs stored in glove box.

Time (h)	V_{oc} (V)	J_{sc} (mA cm^{-2})	*FF* (%)	PCE (%)	Normalized PCE
0 h	0.87	24.50	75.49	16.11	1
24 h	0.86	24.26	74.26	15.57	0.966
48 h	0.86	23.96	72.83	15.08	0.936
96 h	0.86	23.78	71.65	14.72	0.913
120 h	0.86	23.45	70.36	14.24	0.883
168 h	0.85	23.01	69.02	13.50	0.838
216 h	0.84	22.76	67.96	13.04	0.810

Table S3. Mobility data of the PSCs for devices based on PBBD, PBBD-Cl-α and PBBD-Cl-β.

Polymers	μ_h^{max} ($cm^2 v^{-1} s^{-1}$)	μ_e^{max} ($cm^2 v^{-1} s^{-1}$)	μ_h/μ_e
PBBD	6.6×10^{-5} (6.2×10^{-5})[a]	4.2×10^{-5} (3.8×10^{-5})[a]	1.6
PBBD-Cl-α	8.5×10^{-5} (7.8×10^{-5})[a]	5.6×10^{-5} (5.1×10^{-5})[a]	1.5
PBBD-Cl-β	1.6×10^{-4} (1.1×10^{-4})[a]	1.2×10^{-4} (1.0×10^{-4})[a]	1.3

[a] Average values based on 10 devices.

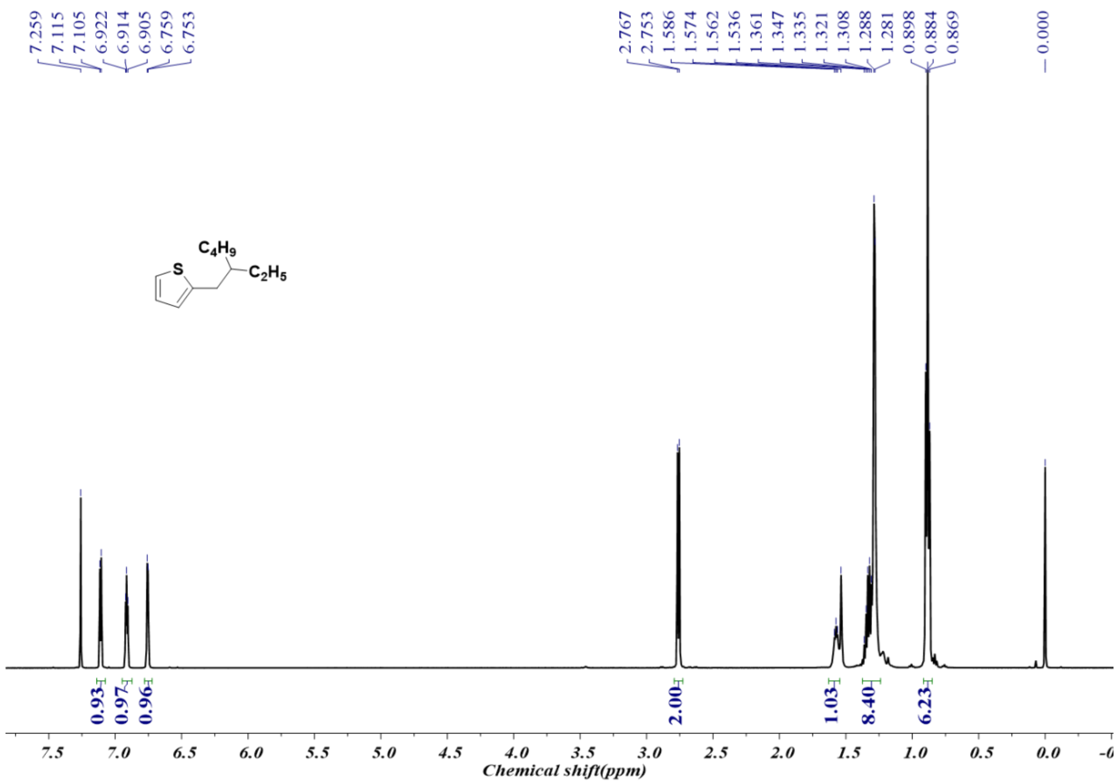

Figure S11. ^{1}H NMR spectrum of compound (3).

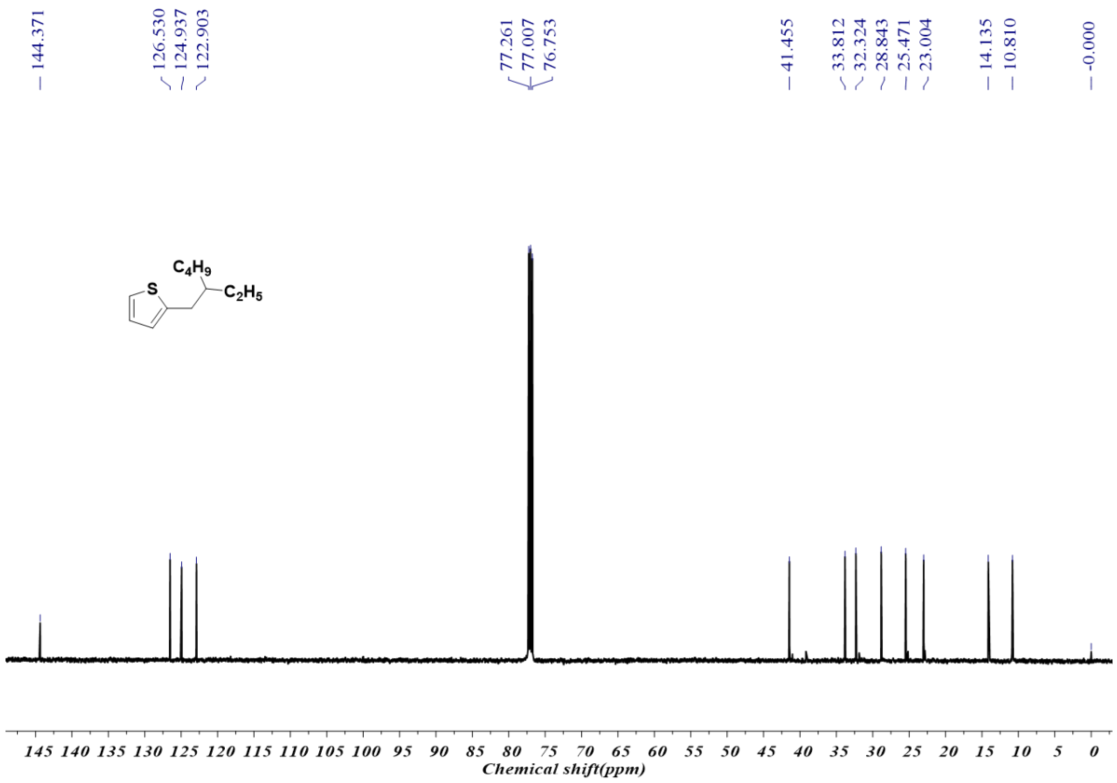

Figure S12. ^{13}C NMR spectrum of compound (3).

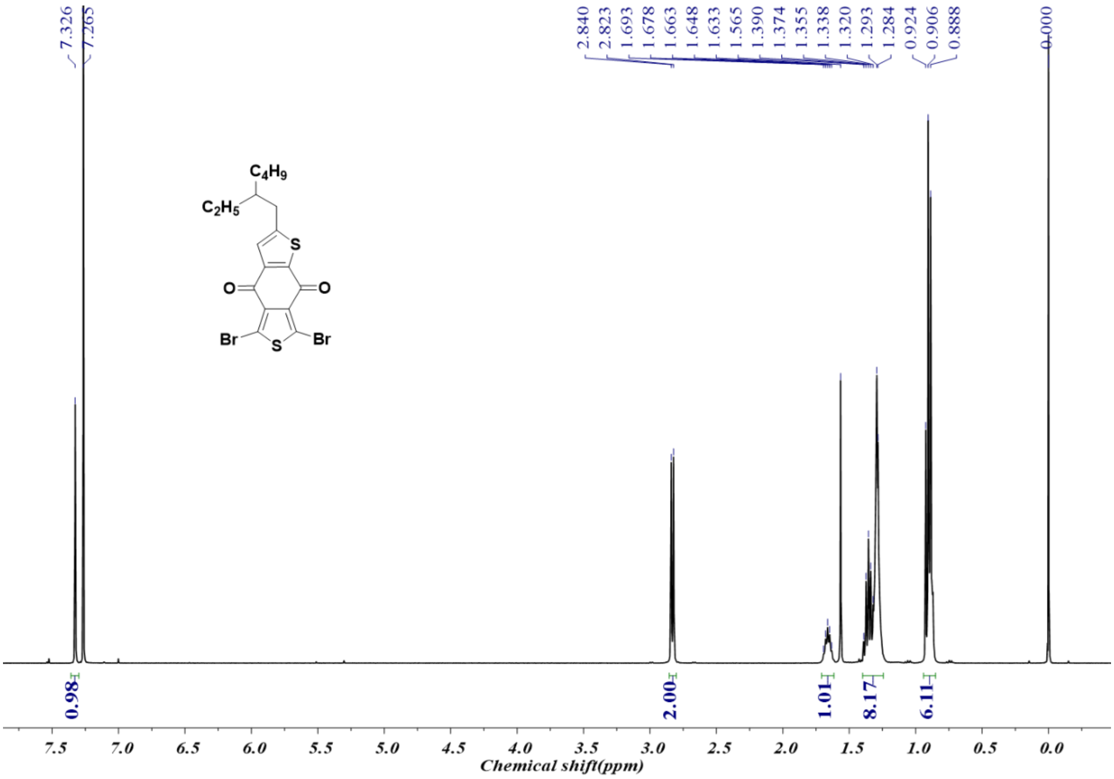

Figure S13. ^{1}H NMR spectrum of compound (6).

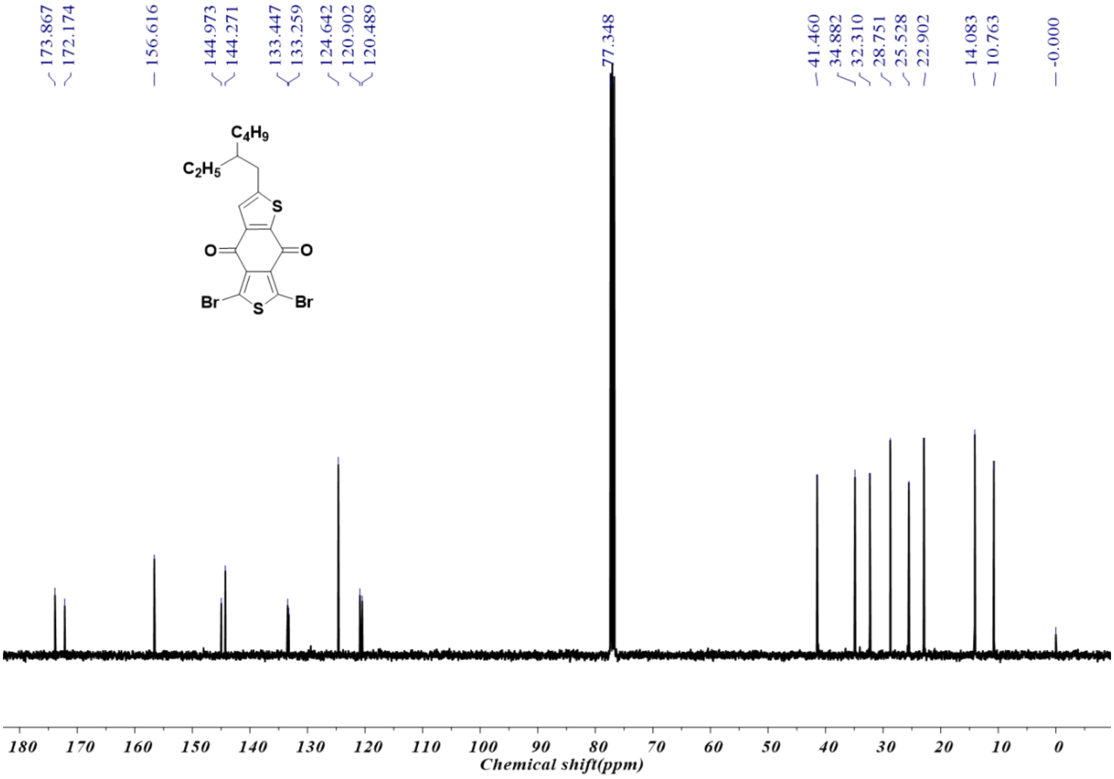

Figure S14. ^{13}C NMR spectrum of compound (6).

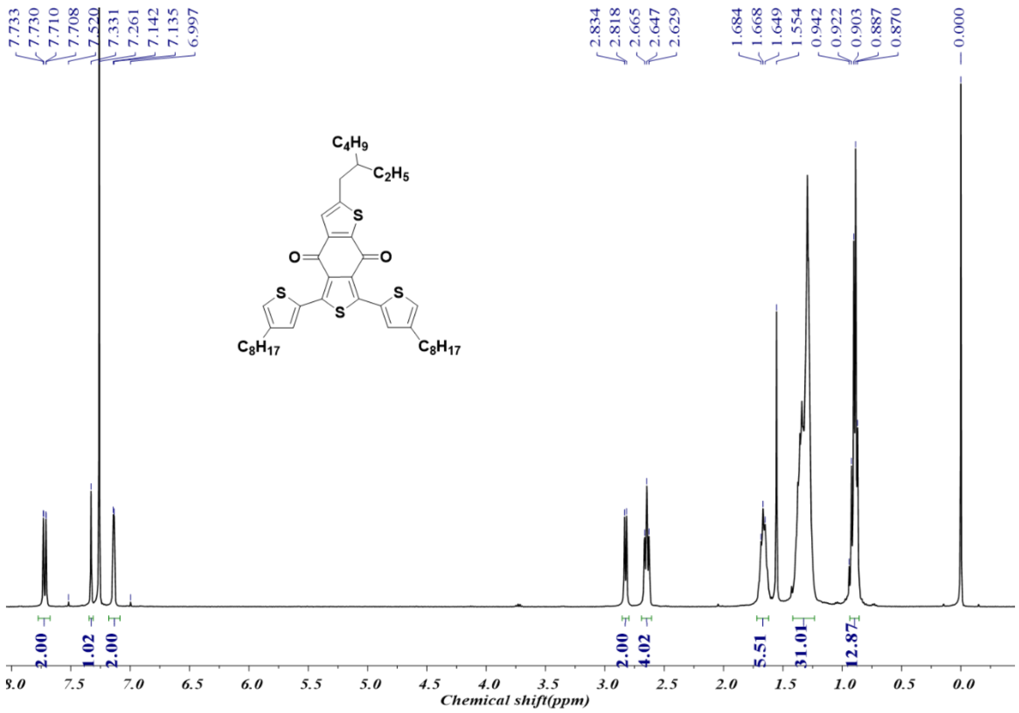

Figure S15. ^{1}H NMR spectrum of compound (9).

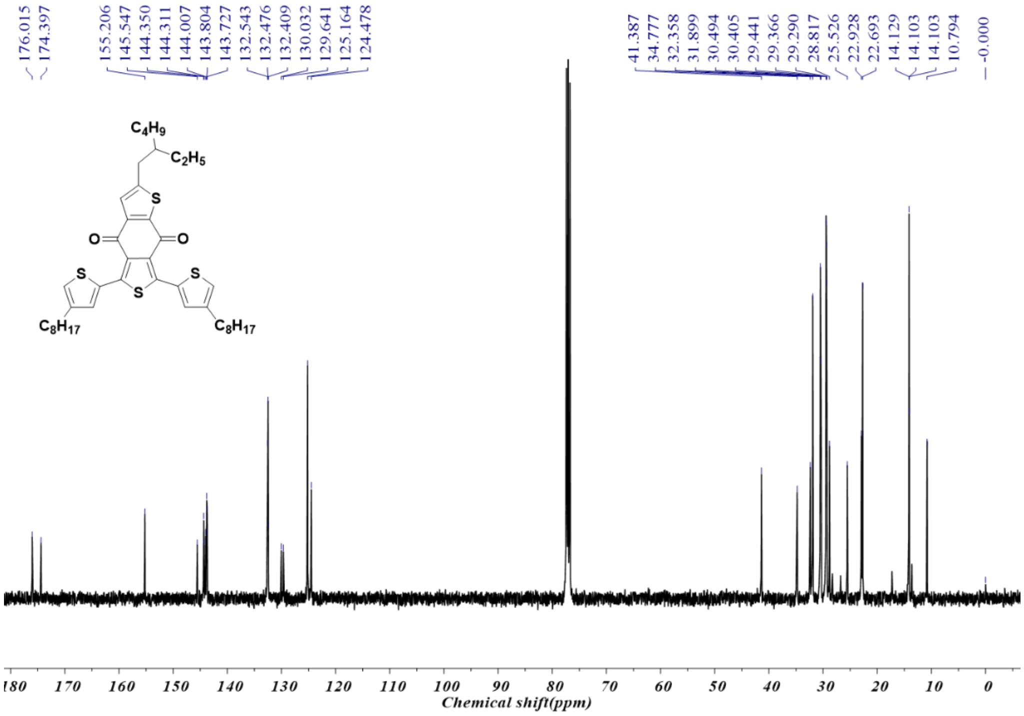

Figure S16. ^{13}C NMR spectrum of compound (9).

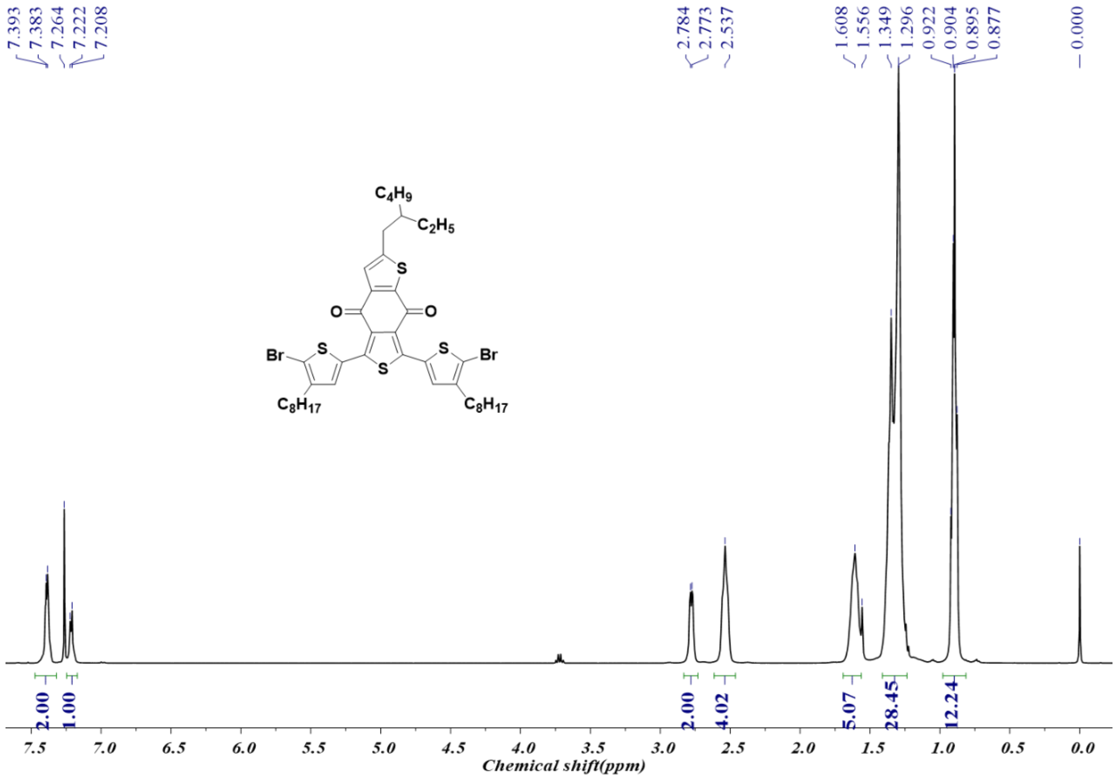

Figure S17. ^{1}H NMR spectrum of compound M1.

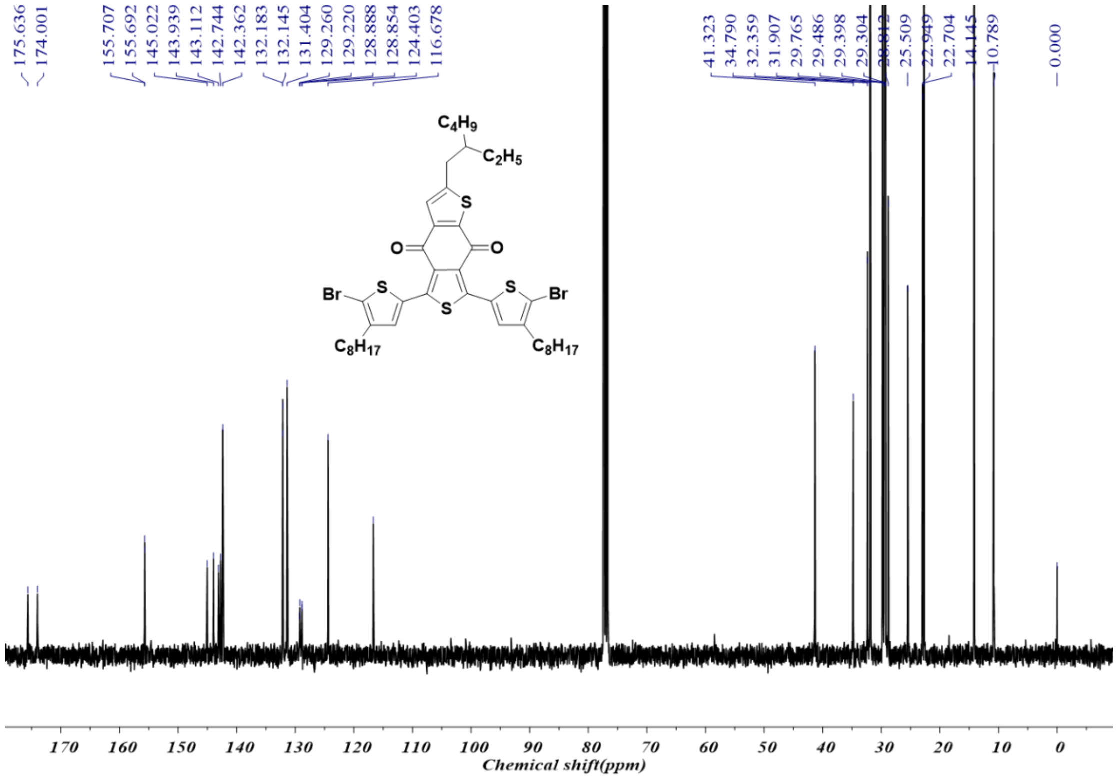

Figure S18. ^{13}C NMR spectrum of compound M1.

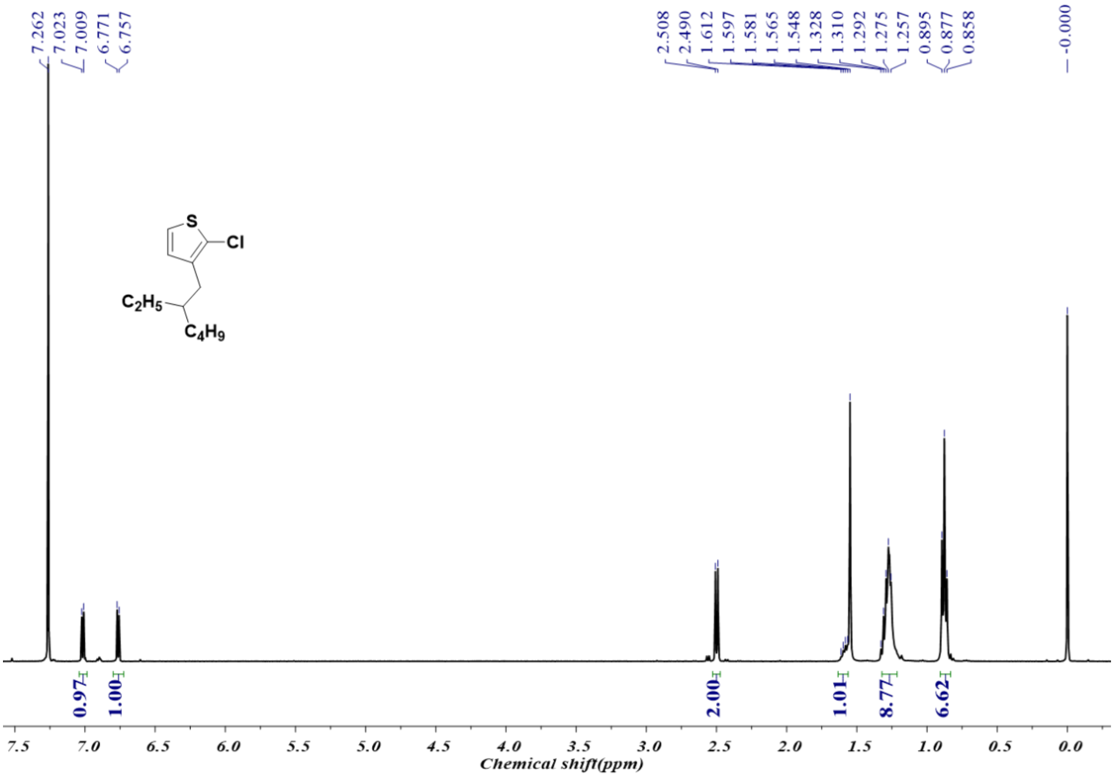

Figure S19. ^{1}H NMR spectrum of compound (4).

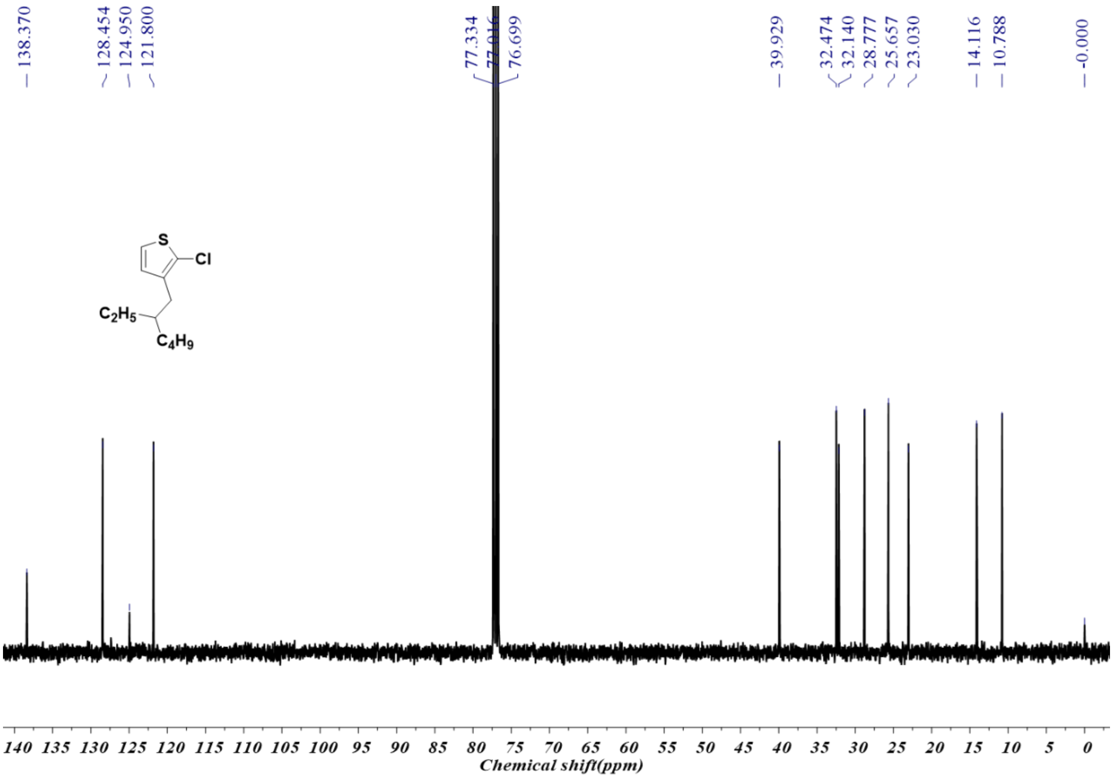

Figure S20. ^{13}C NMR spectrum of compound (4).

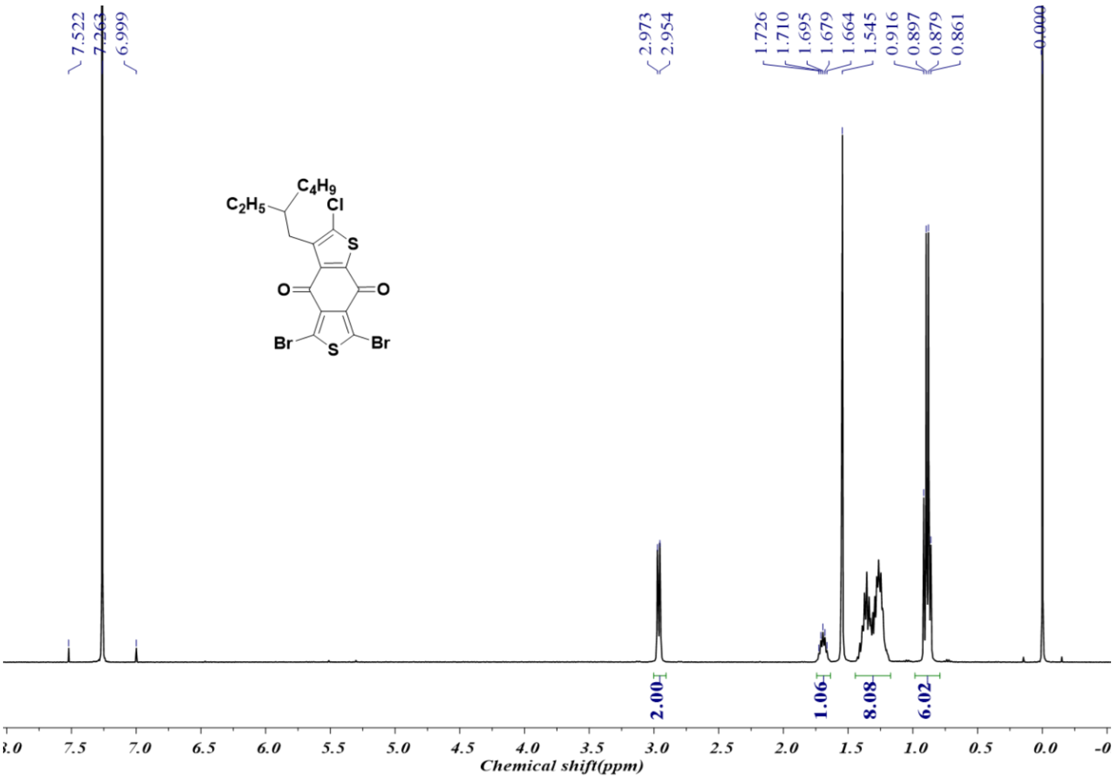

Figure S21. ^{1}H NMR spectrum of compound (7).

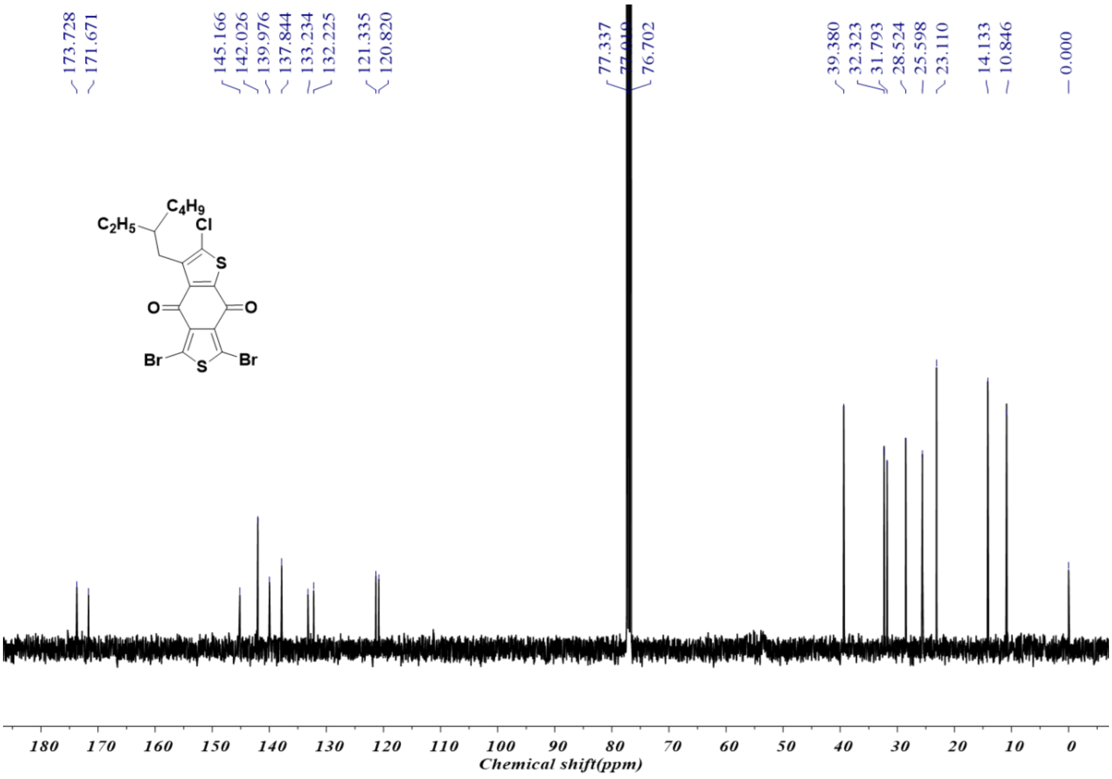

Figure S22. ^{13}C NMR spectrum of compound (7).

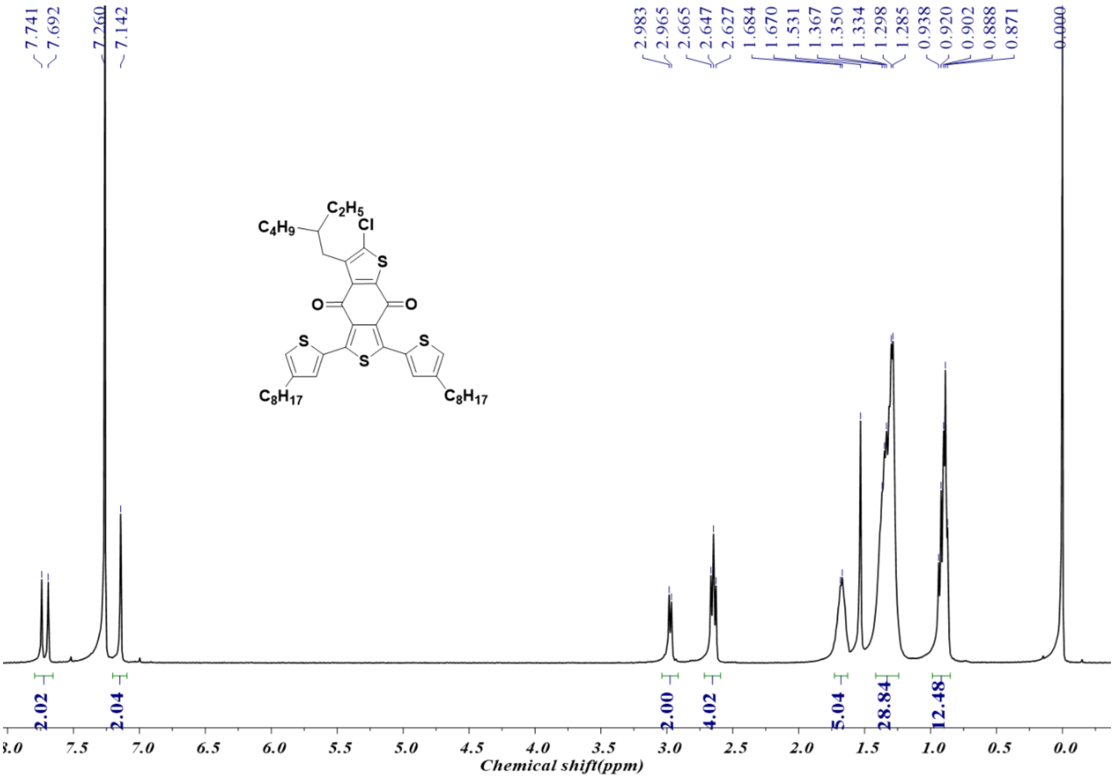

Figure S23. ^{1}H NMR spectrum of compound (10).

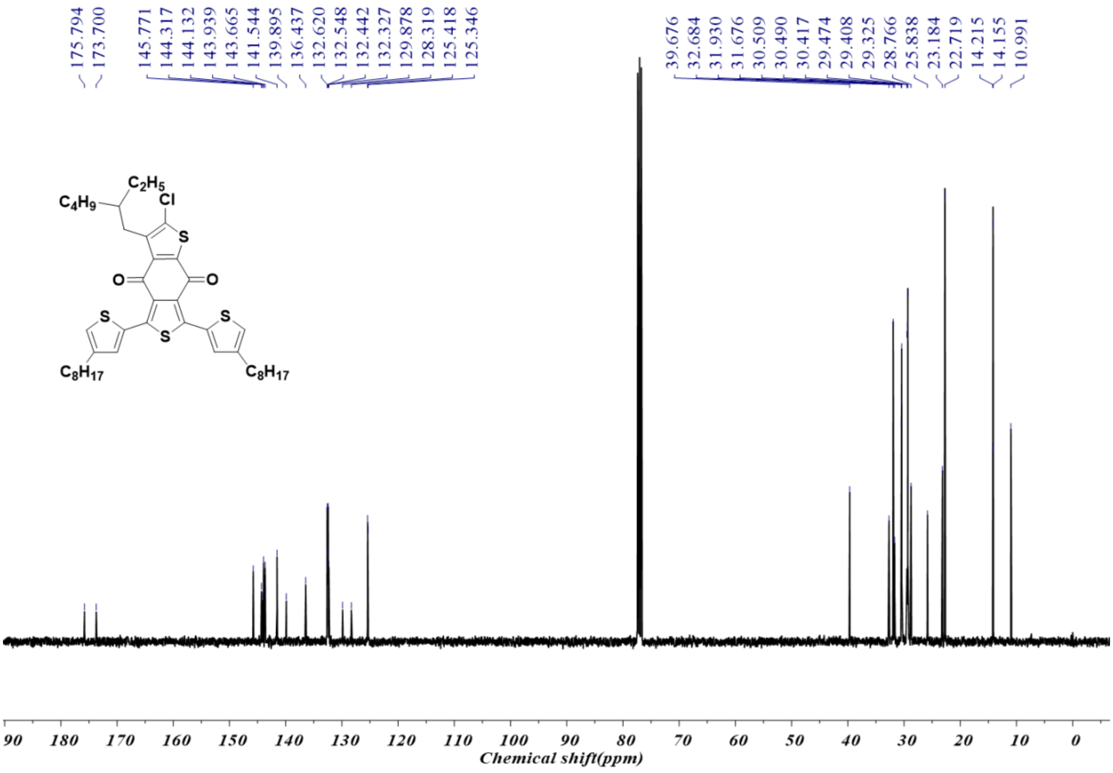

Figure S24. ^{13}C NMR spectrum of compound (10).

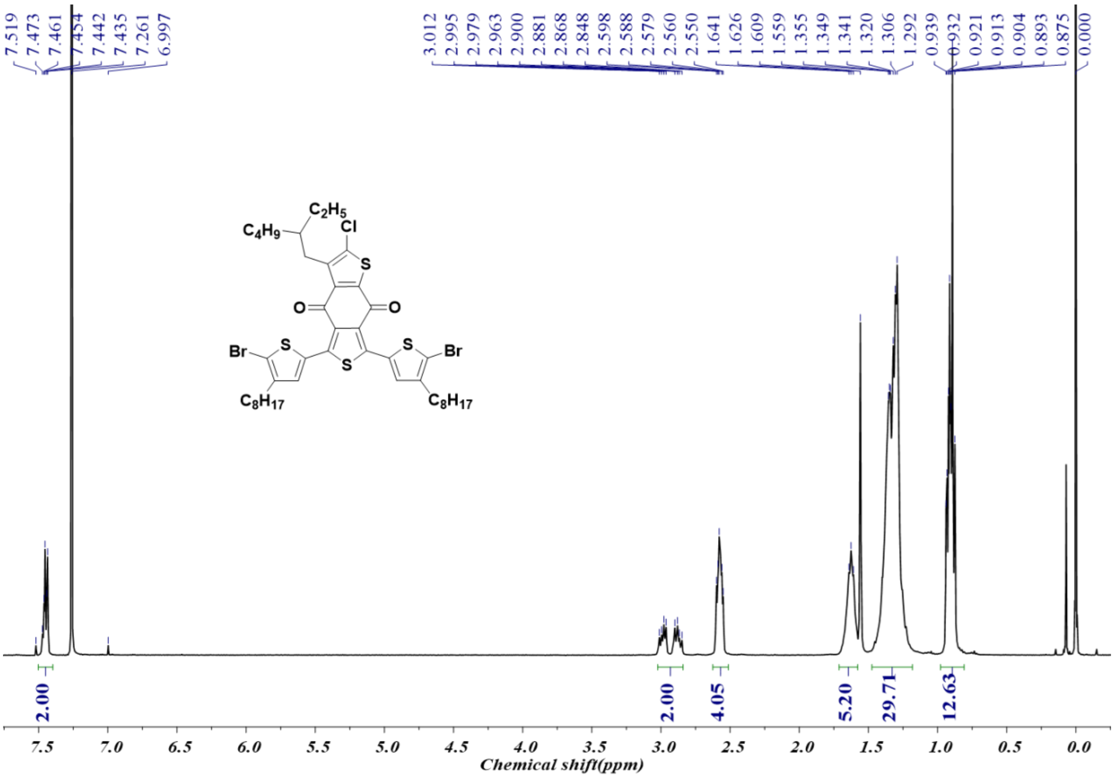

Figure S25. ^{1}H NMR spectrum of compound M2.

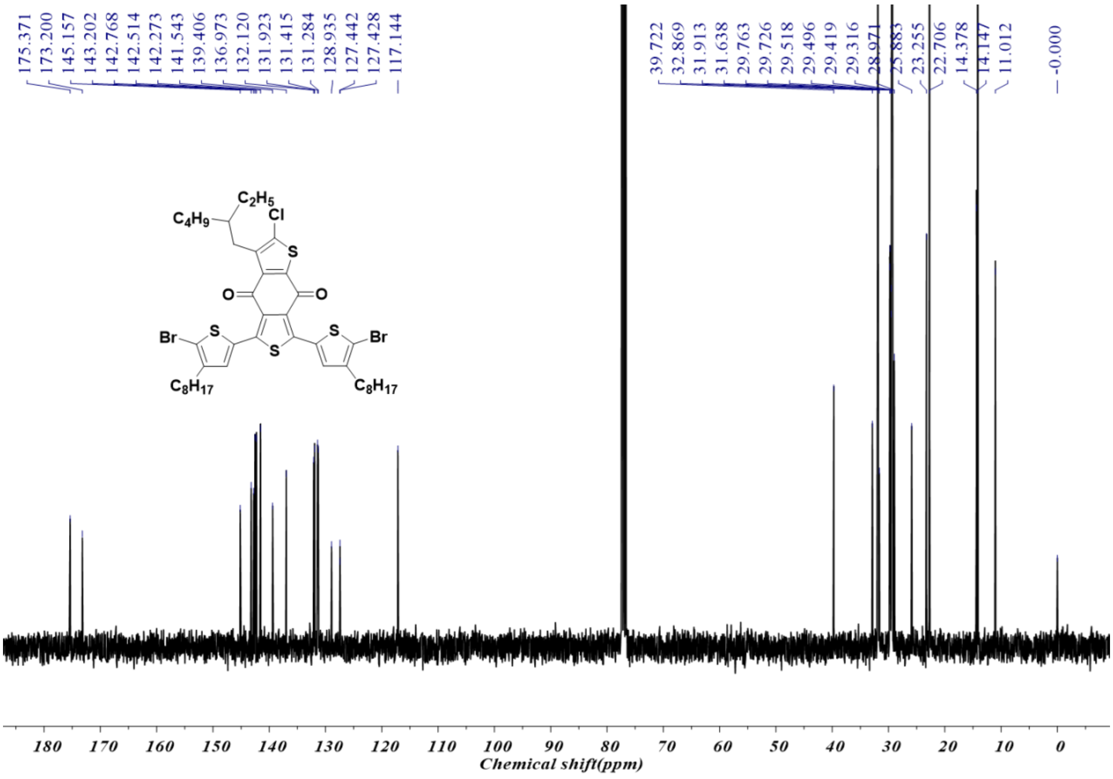

Figure S26. ^{13}C NMR spectrum of compound M2.

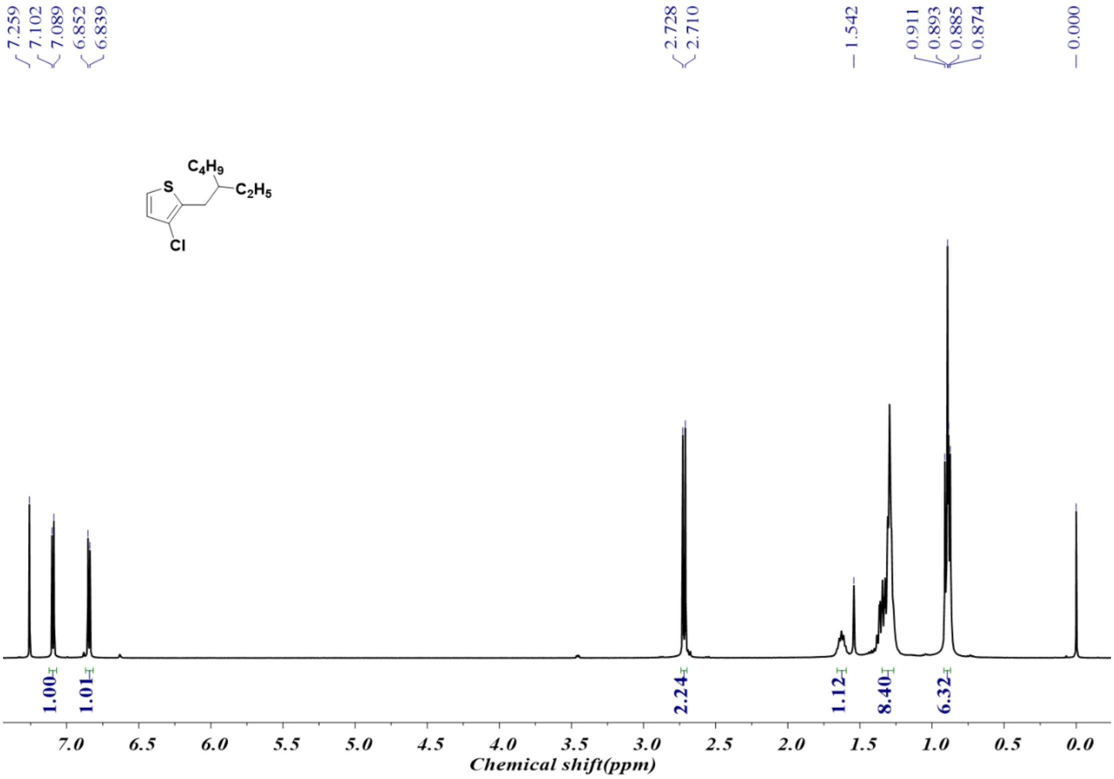

Figure S27. ^{1}H NMR spectrum of compound (5).

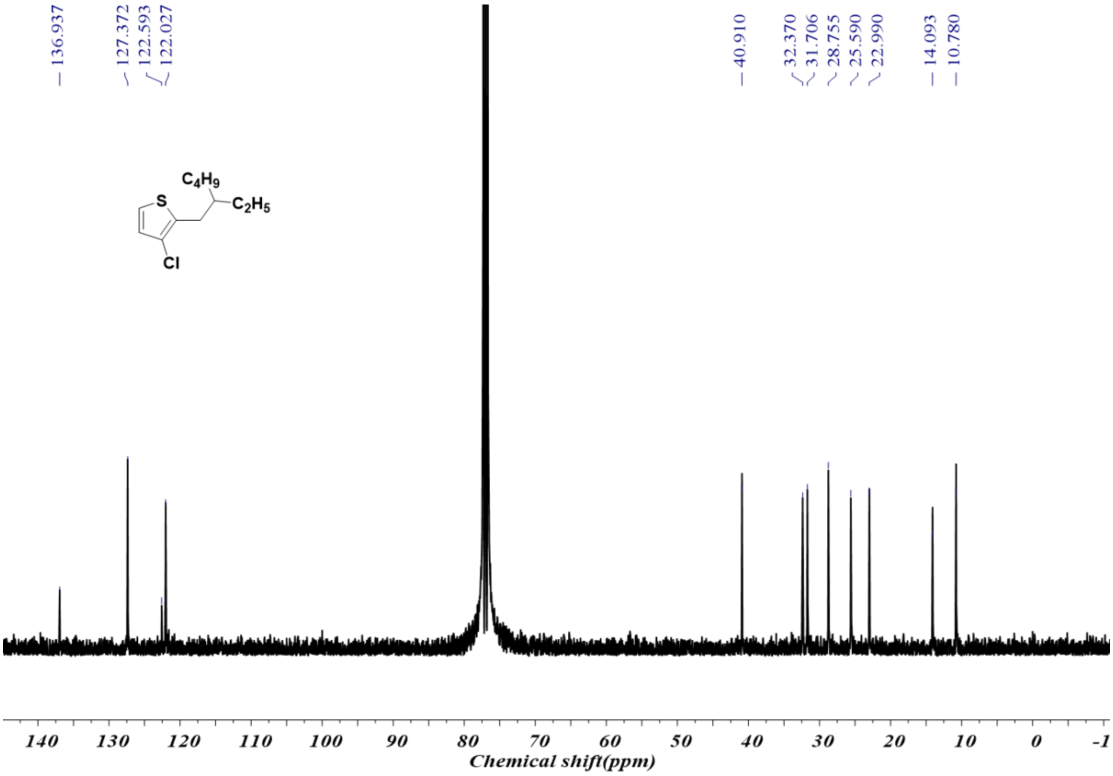

Figure S28. ^{13}C NMR spectrum of compound (5).

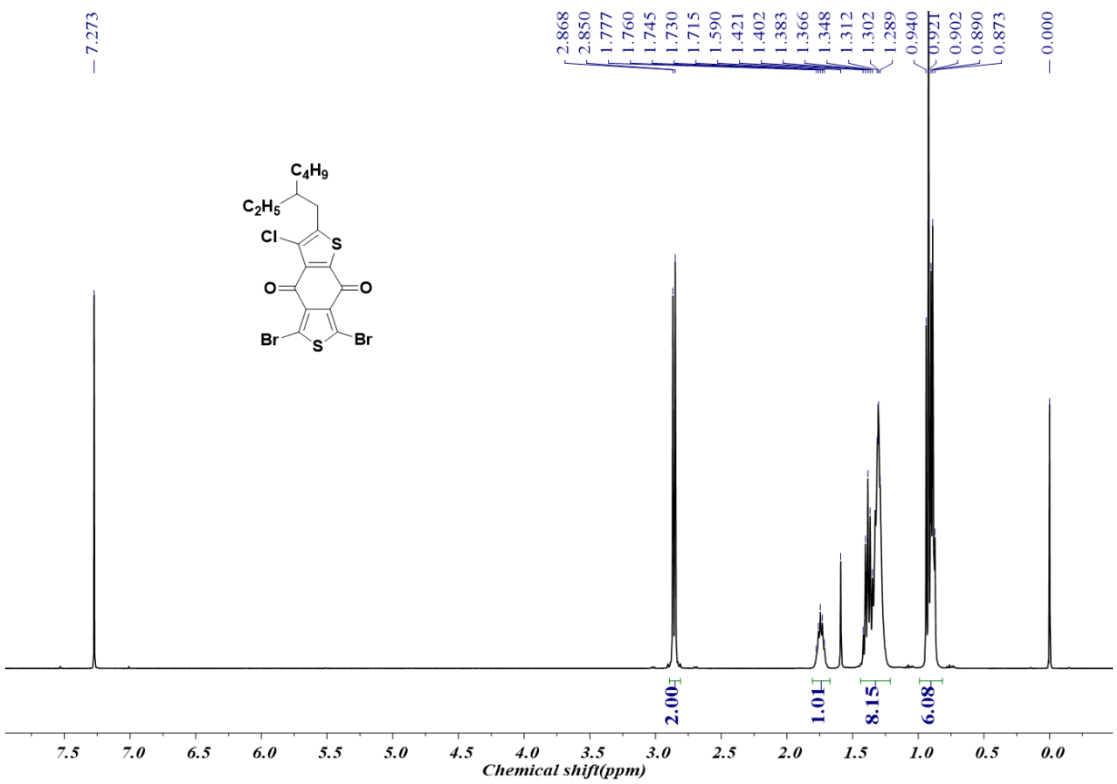

Figure S29. ^{1}H NMR spectrum of compound (8).

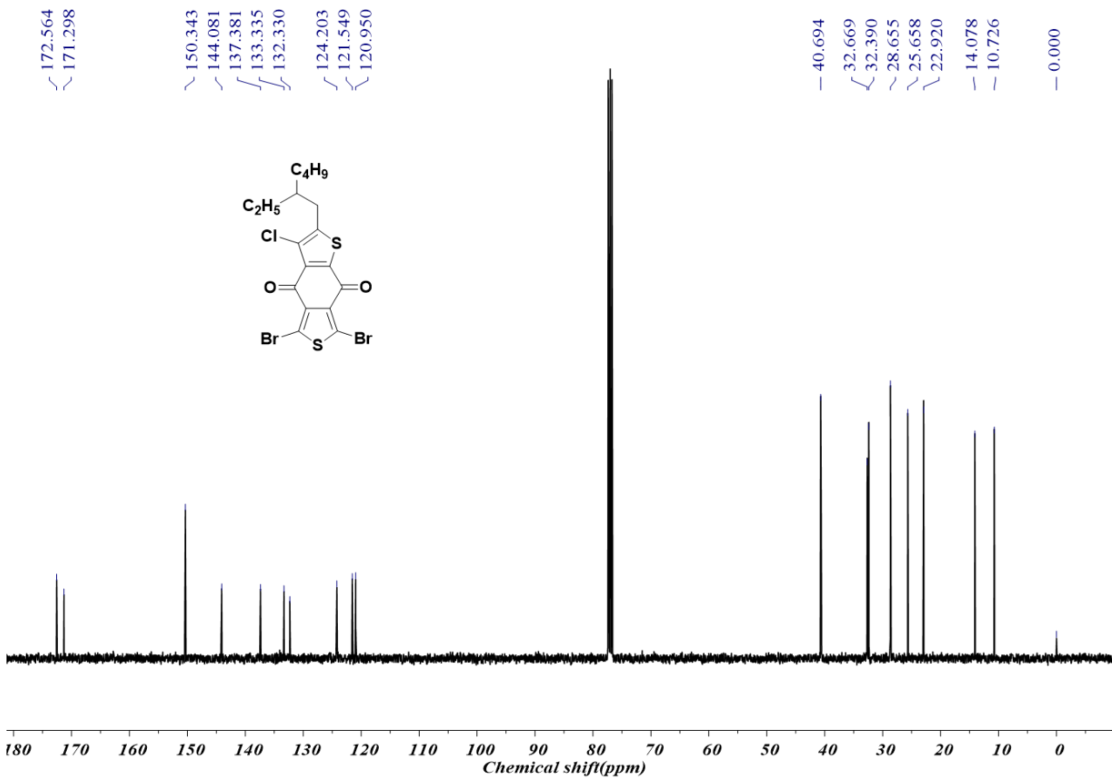

Figure S30. ^{13}C NMR spectrum of compound (8).

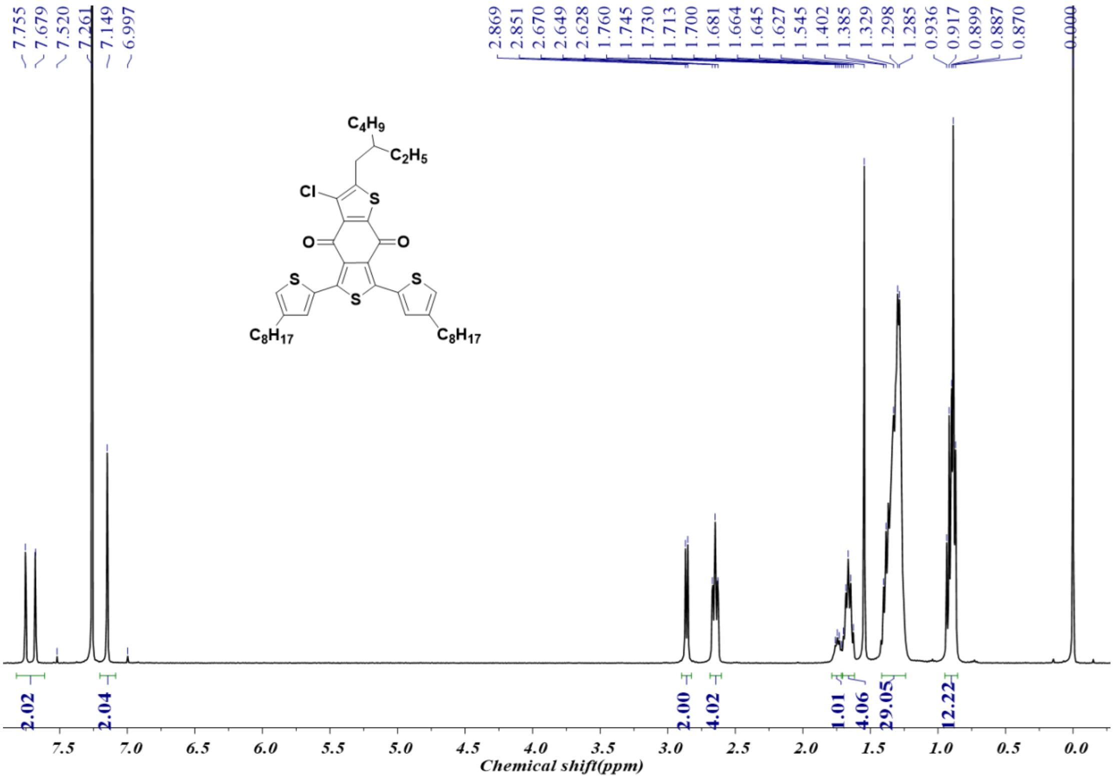

Figure S31. ^{1}H NMR spectrum of compound (11).

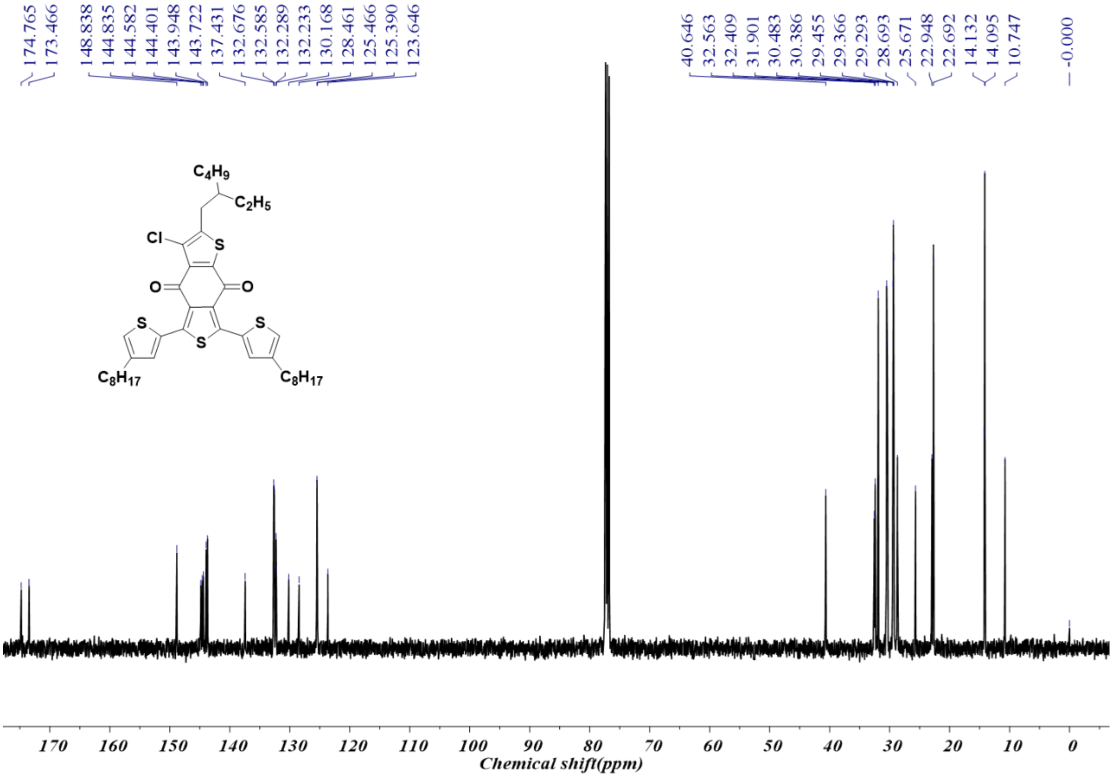

Figure S32. ^{13}C NMR spectrum of compound (11).

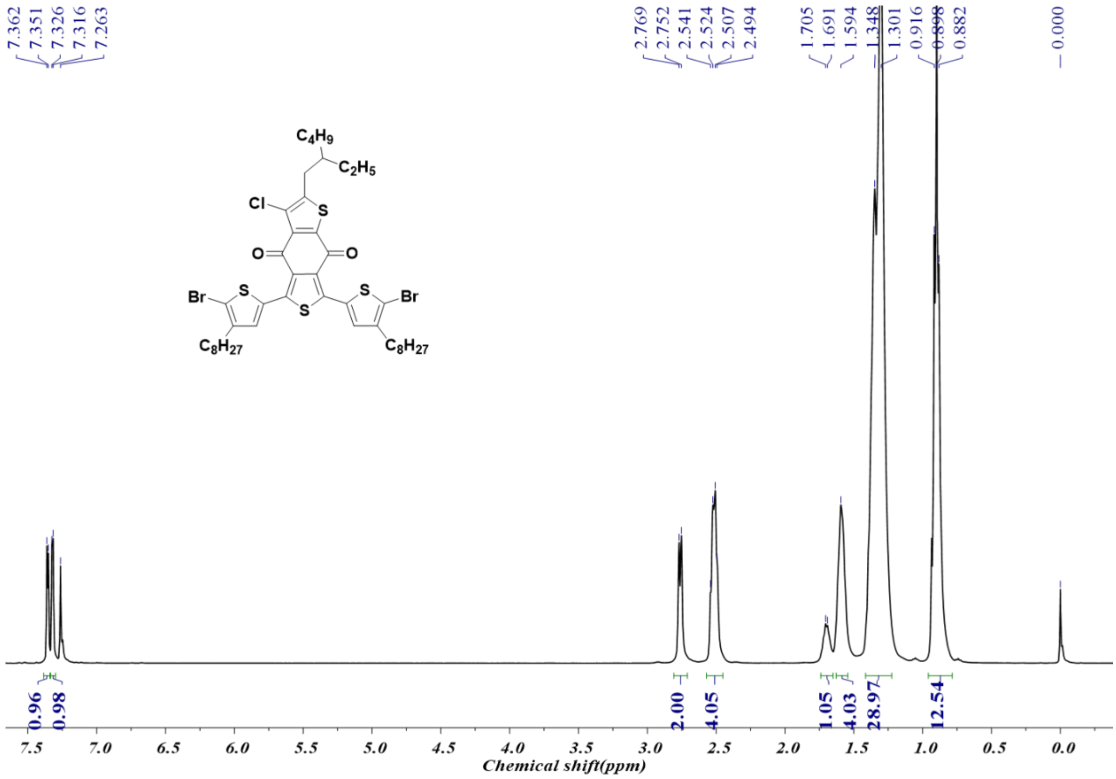

Figure S33. ^{1}H NMR spectrum of compound M3.

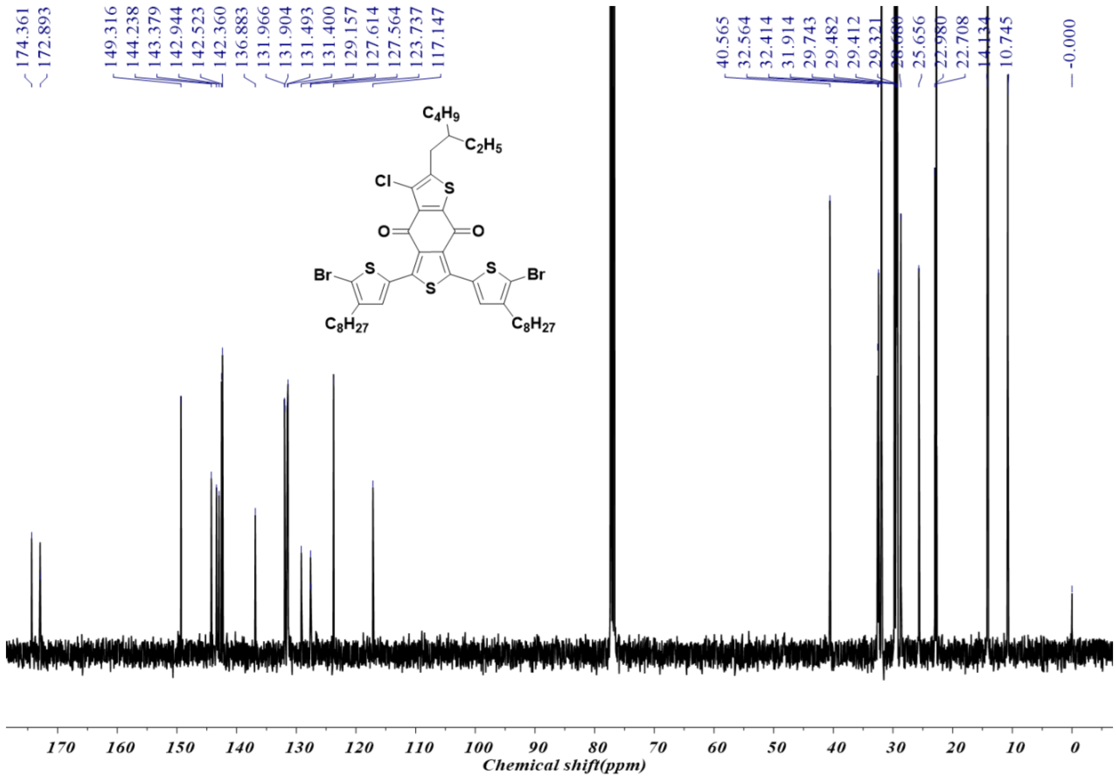

Figure S34. ^{13}C NMR spectrum of compound M3 .

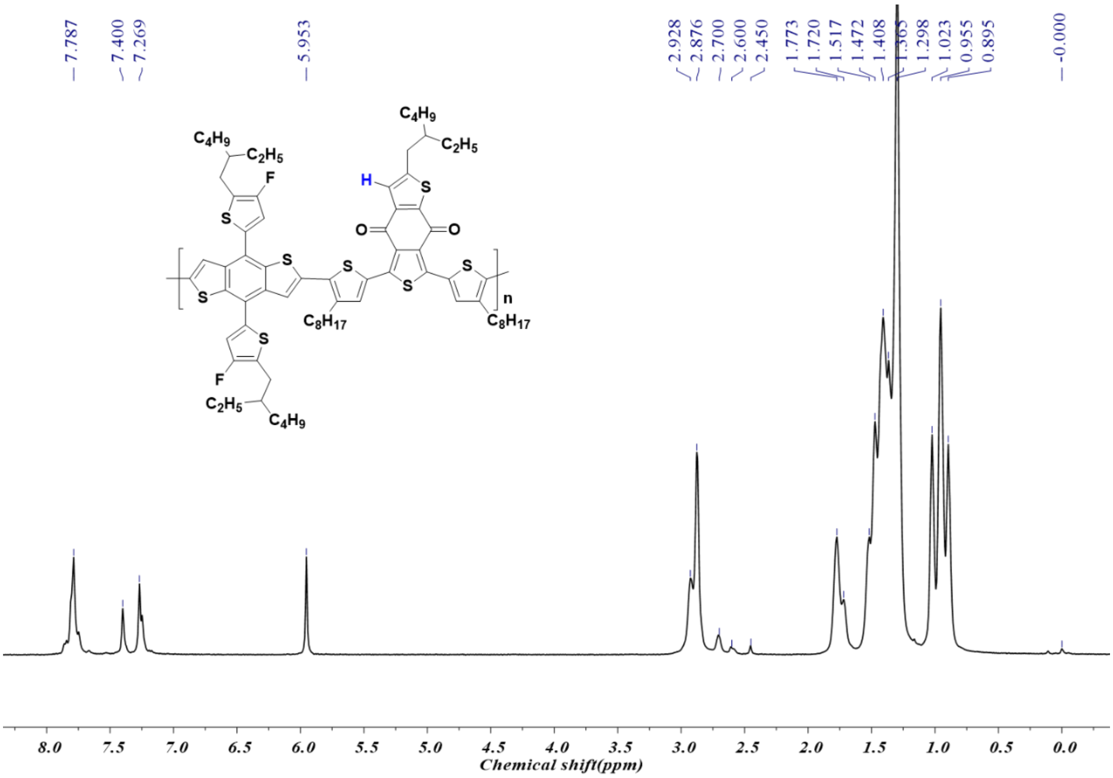

Figure S35. ^{1}H NMR spectrum of polymer PBBD.

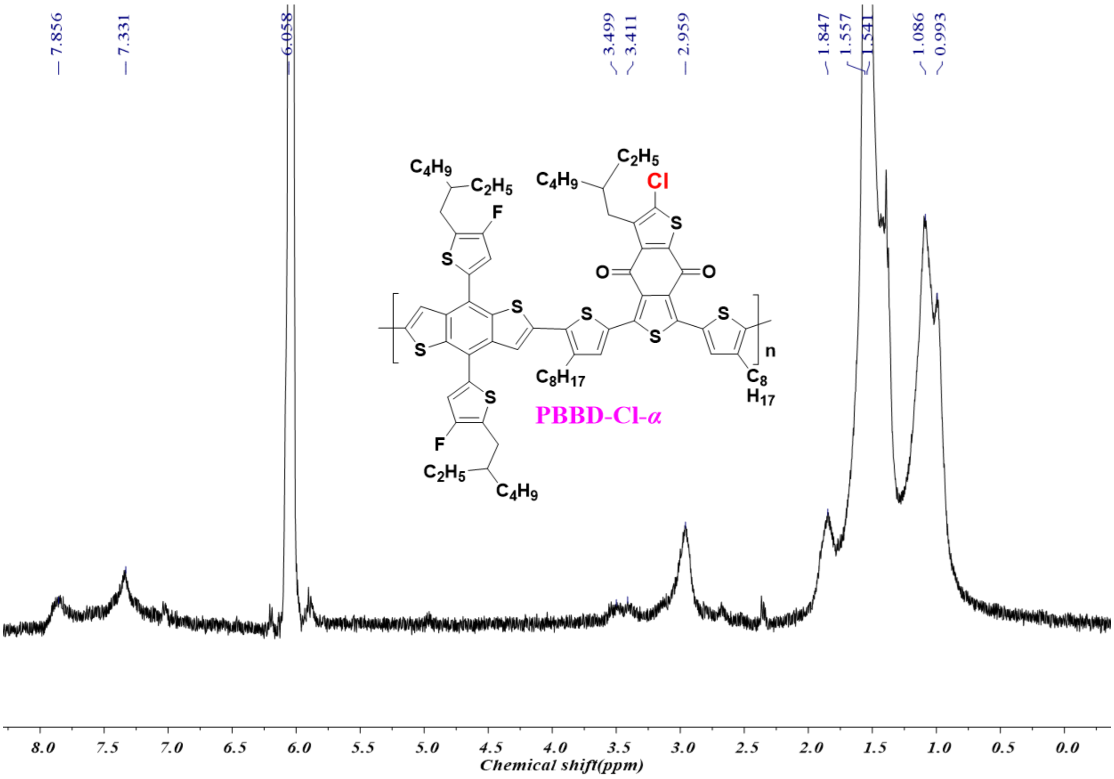

Figure S36. ^{1}H NMR spectrum of polymer PBBD-Cl-*α*.

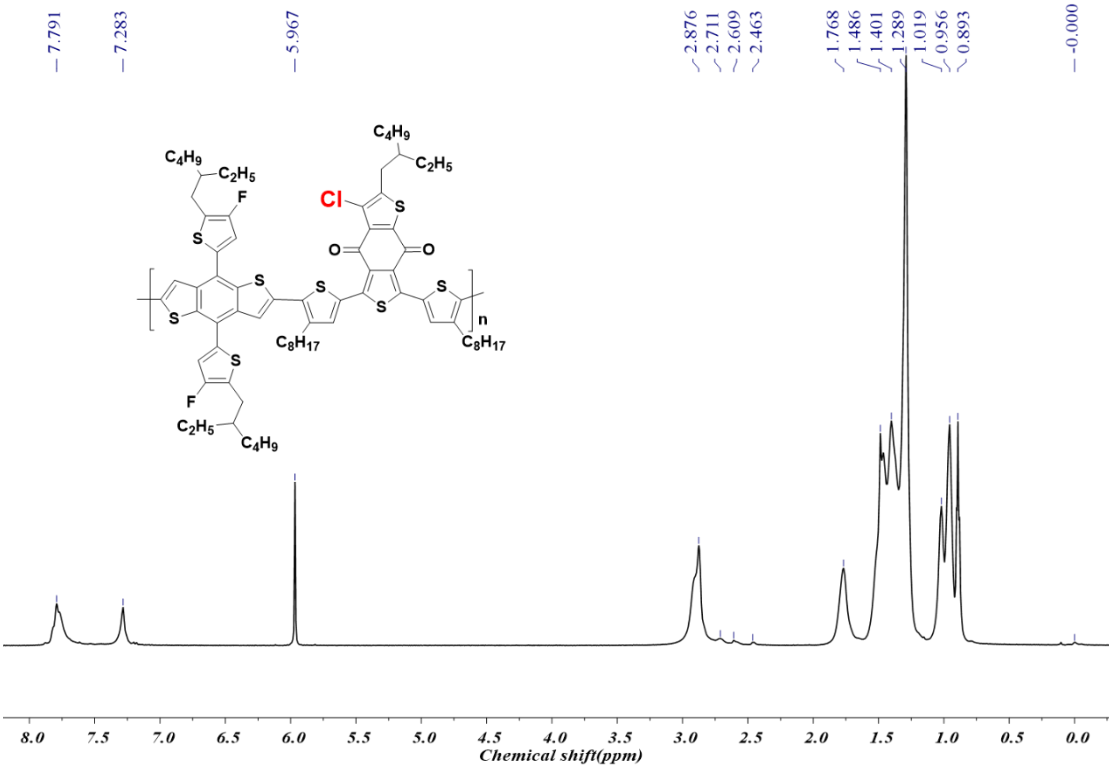

Figure S37. ^{1}H NMR spectrum of compound PBBD-Cl-*β*.

MW Averages

Mp: 64065	Mn: 30600	Mv: 60441	Mw: 66282
Mz: 111296	Mz+1: 159052	PD: 2.1661	

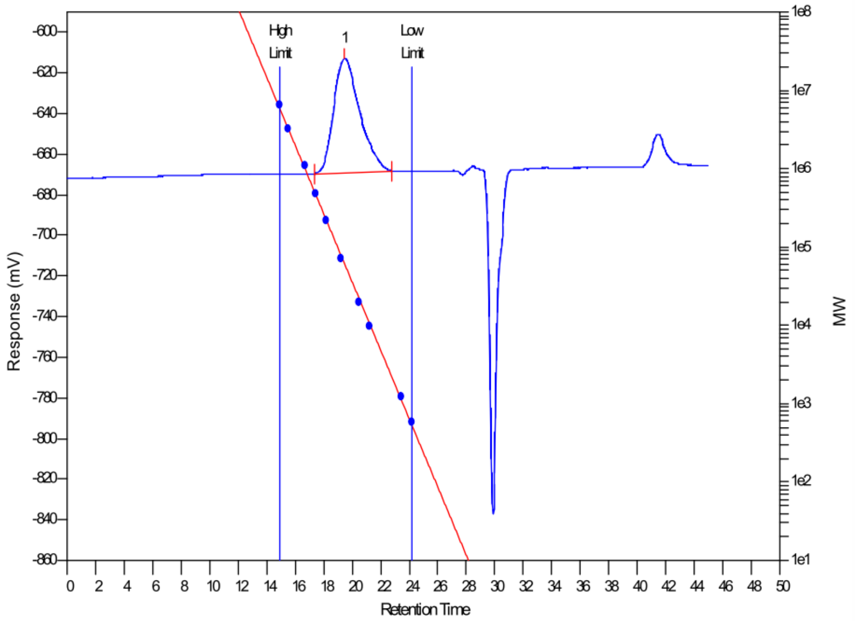

Figure S38. GPC data of polymer PBBD.

MW Averages

Mp: 77007 Mn: 33529 Mv: 72065 Mw: 79438

Mz: 136362 Mz+1: 197232 PD: 2.3692

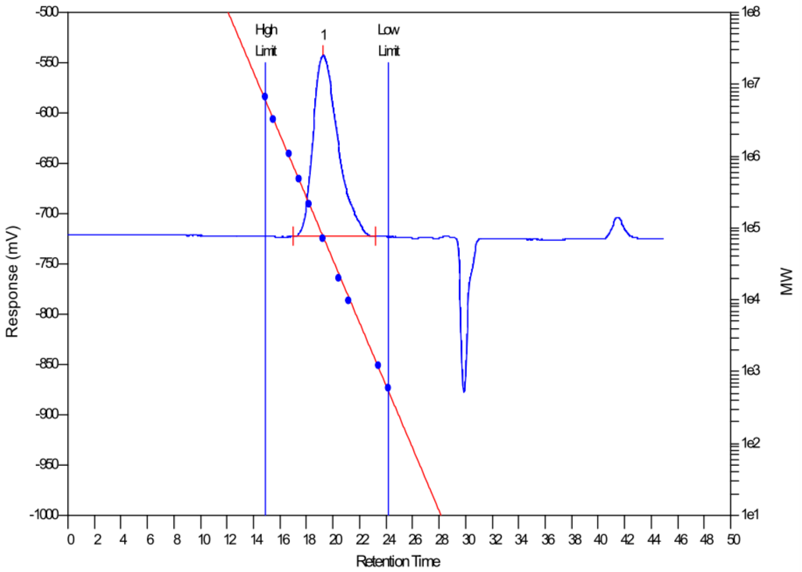

Figure S39. GPC data of polymer PBBD-Cl-α.

MW Averages

Mp: 49021 Mn: 31067 Mv: 52502 Mw: 56903

Mz: 91333 Mz+1: 128905 PD: 1.8316

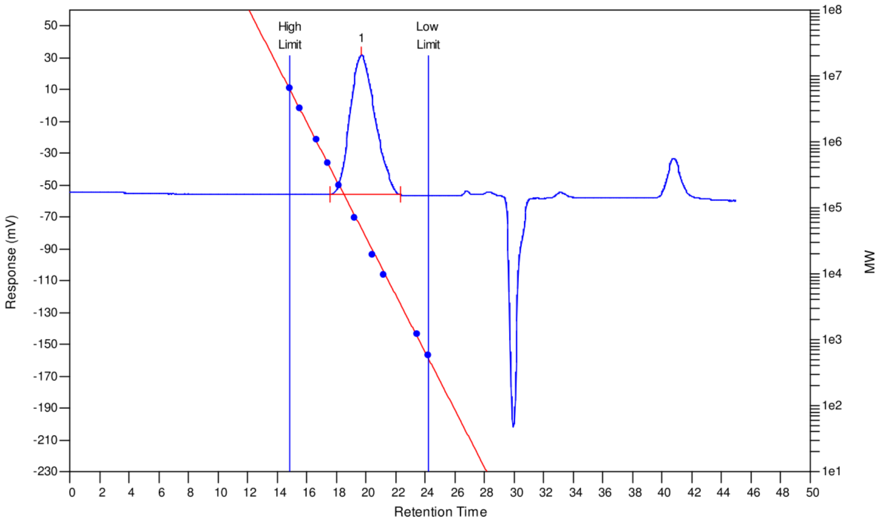

Figure S40. GPC data of polymer PBBD-Cl-β.
